# Single-molecule fluorescence detection of a tricyclic nucleoside analogue†

**DOI:** 10.1039/d0sc03903a

**Published:** 2020-12-28

**Authors:** George N. Samaan, Mckenzie K. Wyllie, Julian M. Cizmic, Lisa-Maria Needham, David Nobis, Katrina Ngo, Susan Andersen, Steven W. Magennis, Steven F. Lee, Byron W. Purse

**Affiliations:** Department of Chemistry and Biochemistry and the Viral Information Institute, San Diego State University San Diego CA 92182 USA bpurse@sdsu.edu; University of Cambridge, Chemistry Department Lensfield Road Cambridge CB2 1EW UK; School of Chemistry, University of Glasgow University Avenue Glasgow G12 8QQ UK

## Abstract

Fluorescent nucleobase surrogates capable of Watson–Crick hydrogen bonding are essential probes of nucleic acid structure and dynamics, but their limited brightness and short absorption and emission wavelengths have rendered them unsuitable for single-molecule detection. Aiming to improve on these properties, we designed a new tricyclic pyrimidine nucleoside analogue with a push–pull conjugated system and synthesized it in seven sequential steps. The resulting *C*-linked 8-(diethylamino)benzo[*b*][1,8]naphthyridin-2(1*H*)-one nucleoside, which we name ABN, exhibits *ε*_442_ = 20 000 M^−1^ cm^−1^ and *Φ*_em,540_ = 0.39 in water, increasing to *Φ*_em_ = 0.50–0.53 when base paired with adenine in duplex DNA oligonucleotides. Single-molecule fluorescence measurements of ABN using both one-photon and two-photon excitation demonstrate its excellent photostability and indicate that the nucleoside is present to > 95% in a bright state with count rates of at least 15 kHz per molecule. This new fluorescent nucleobase analogue, which, in duplex DNA, is the brightest and most red-shifted known, is the first to offer robust and accessible single-molecule fluorescence detection capabilities.

## Abstract 2

Fluorescent nucleoside analogue ABN is readily detected at the single-molecule level and retains a quantum yield >50% in duplex DNA oligonucleotides.

## Graphical abstract

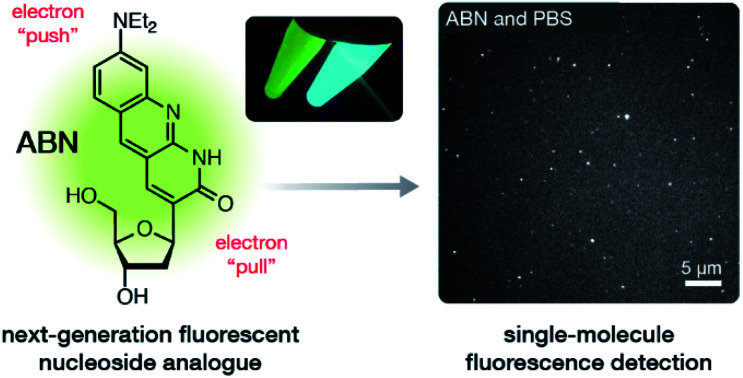

## Introduction

Single-molecule fluorescence studies of biological molecules have a unique capacity to provide mechanistic insights into the relationships between structural dynamics and function, which are lost to averaging in ensemble measurements.^1–3^ Most of these studies have used extrinsic fluorophores, which can potentially interfere with the native biomolecular behavior and obscure local structural details.^4–7^ An ideal approach in this regard would be the use of intrinsically fluorescent biomolecules, prepared by the synthetic introduction of only minimal changes.^8^ It remains, however, a major challenge to attain adequate brightness and photostability in this approach.

Fluorescent nucleobase analogues (FBAs) have been a mainstay of biophysical studies of nucleic acid structure and dynamics because they can be placed precisely in a desired sequence and are less structurally perturbing than proximally tethered fluorophores.^9,10^ They are available with a range of fluorescent properties and, in response to base pairing and stacking, their fluorescence may be quenched (*e.g.* 2-aminopurine), retained (*e.g.* tC and ^th^G) or turned on (*e.g.*^DEA^tC).^11–14^ However, few nucleobase analogues have extinction coefficients >10^4^ with *Φ*_em_ > 0.3; most are approximately an order of magnitude dimmer than conventional fluorophores such as Alexa Fluor 488 and rhodamine B.^8,9,15–17^ This lack of brightness has rendered them largely unsuitable for single-molecule fluorescence studies.^18–20^ Furthermore, with the current rapid development of spatially-resolved transcriptomics and genomics, there is clearly a future need for fluorescent analogues that can act as effective single-molecule probes.^21,22^

Conventional fluorophores typically exhibit superior optical properties to fluorescent nucleobase analogues, primarily because of their larger extinction coefficients—sometimes in excess of 10^5^—but they need not be larger molecules.^23,24^ In structures such as rhodamine B, there is a prominent push–pull character, but the benzenecarboxylic acid is twisted out of plane and does not contribute significantly to the photophysical properties. The fluorescent, tricyclic core is similar in size to many of the common fluorescent nucleotides, but the most important difference is that push–pull motifs are underrepresented in existing FBAs.^8,9,25^ The required positions of heteroatoms for the Watson–Crick face and the glycosidic bond make incorporation of this motif challenging.

**Fig. 1:**
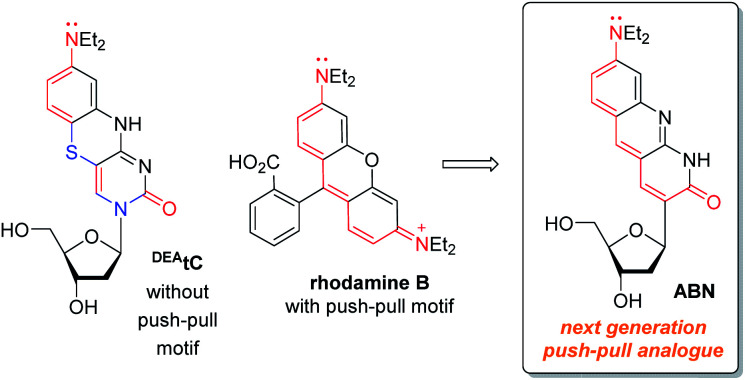
Push–pull motifs are hallmarks of bright organic fluorophores, but are rare in fluorescent nucleosides. The redesign of a nucleoside analogue to include this motif significantly increases *ε* and *Φ*_em_, enabling single-molecule detection.

In this work, we hypothesized that, by redesigning a fluorescent, tricyclic cytidine analogue to include a push–pull motif, a substantial enhancement of brightness could be obtained (Fig. 1). A comparison of ^DEA^tC with rhodamine B shows that the electronic nature of two heteroatoms—S and the glycosidic N—differentiate ^DEA^tC from a more conventional push–pull fluorophore.^13,14^ A structure redesigned by replacing both atoms with sp^2^ C would impart the nucleoside analogue with a push–pull character, while maintaining the capacity for Watson–Crick hydrogen bonding and without further structural perturbation. The resulting nucleoside analogue, whose synthesis and characterization we report here, includes 8-(diethylamino)benzo[*b*][1,8]naphthyridin-2(1*H*)-one as a fluorescent nucleobase surrogate, and we name this new compound ABN. Single-molecule fluorescence measurements show that the compound exists to >95% in a bright state and can be detected using both one- and two-photon excitation.

## Results and discussion

### Synthesis

**Fig. 2:**
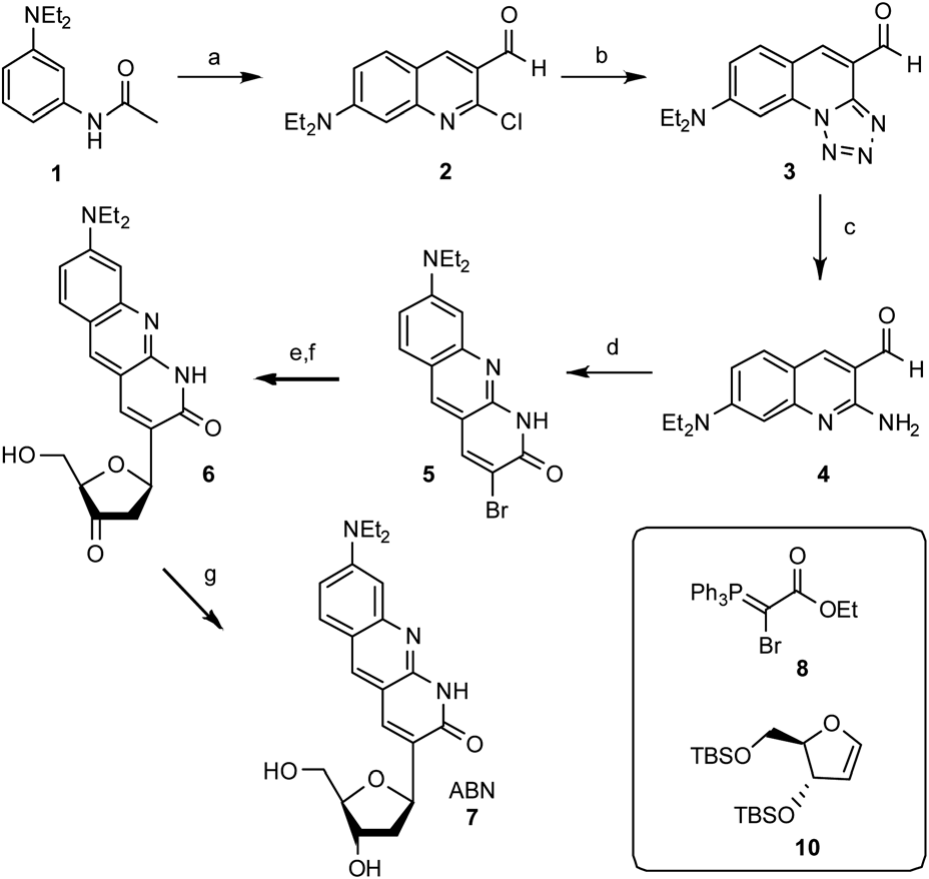
Synthesis of the ABN nucleoside analogue. Reagents, conditions, and yields: (a) DMF, POCl_3_, 50 °C, 20 min (15%). (b) NaN_3_, DMF, 90 °C, 18 h (85%). (c) PPh_3_, 2 N HCl, reflux, 2 h (70%). (d) 8, NaOEt, ethanol, 70 °C, 4 h. (e) 10, AsPh_3_, Pd(OAc)_2_, Bu_3_N, 60 °C, 18 h. (f) TBAF, AcOH, rt, 1 h. (g) NaBH(OAc)_3_, AcOH, CH_3_CN, 0 °C, 1 h (9% over 4 steps).

The synthesis of ABN starts with the construction of the bicyclic ring 2-chloro-7-(diethylamino)quinoline-3-carbaldehyde 2 by the reaction of 3-(diethylamino)acetanilide with the Vilsmeier reagent.^26^ The electron donating nature of the diethylamino group renders selective formation of the singly formylated product difficult to achieve, but careful temperature control allows for an adequate yield at multi-gram scale. The quinoline ring 2 undergoes S_N_Ar and cyclization with sodium azide to give a tricyclic compound 3 and the tetrazole ring is then opened reductively by triphenylphosphine in 2 N HCl at reflux to give 2-amino-7-(diethylamino)quinoline-3-carbaldehyde 4. Adding the Wittig reagent ethyl 2-bromo-2-(triphenylphosphoranylidene)acetate 8 (synthesized by the bromination of ethyl (triphenylphosphoranylidene)acetate) to compound 4 yields the brominated tricyclic nucleobase precursor 5.^27^ A Heck reaction of 5 with 3′,5′-*O*-TBS dihydrofuran 10 using palladium acetate and triphenylarsine followed by desilylation with acidic tetrabutylammonium fluoride gives 3′-keto nucleoside 6.^28,29^ This Heck reaction is selective for the β face, owing to the steric influence of the 3′-*O*-TBS group, as usual in the synthesis of *C*-ribosides.^30^ Stereoselective reduction of 6 with sodium triacetoxyborohydride completes the ABN nucleoside 7.^31^ The β configuration of the anomeric *C* is verified by its hydrogen's coupling constants ^3^*J*_H,H_ = 5.9 and 10.0 Hz.^32,33^ A comparison of ^13^C NMR shifts—the carbonyl at *δ* = 165.0 ppm for ABN in CD_3_OD is especially diagnostic—with published data for simpler 1,8-naphthyridin-2(1*H*)-one nucleoside analogues indicates that ABN is present only in the thymidine-like tautomeric form as shown (Fig. 1), to the limit of detection by NMR.^29,34^ Computational prediction of the NMR spectra (MP2/cc-pVDZ/COSMO) confirms this assignment (see ESI†). B3LYP and MP2 calculations using three different basis sets, with and without solvation, predict the T-like tautomer to be 10.3–13.8 kcal mol^−1^ more stable than the cytidine-like tautomer (see the ESI† for details and a molecular model). Dimethoxytritylation followed by 3′-phosphoramidite installation under standard conditions prepares the nucleotide for solid-phase oligonucleotide synthesis (Fig. 2; see the ESI†).

### Oligonucleotide design and preparation

**Fig. 3:**
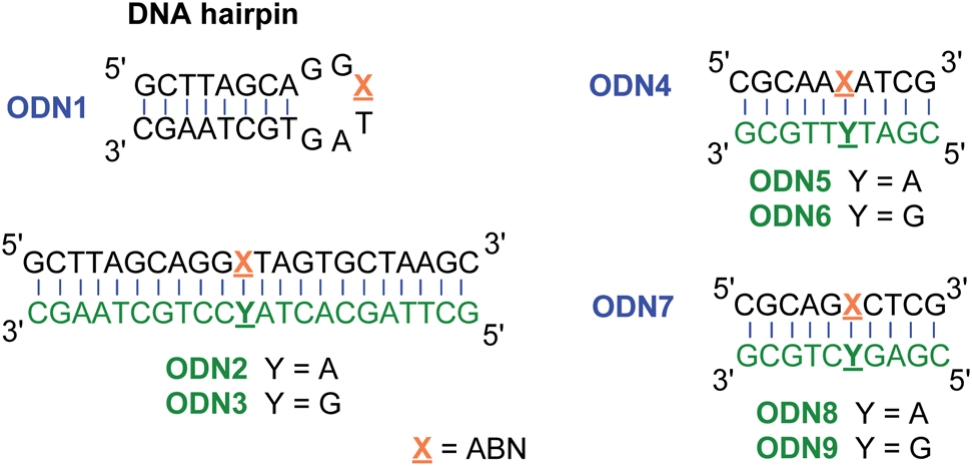
Oligonucleotides used to study the fluorescence and base pairing properties of ABN.

To assess ABN's photophysical properties and natural base mimicry in oligonucleotides, we designed and prepared a hairpin ODN1 and two 10-mer sequences ODN4 and ODN7 that provide a representative set of local environments (Fig. 3). The hairpin was designed to place ABN at the third position of a six-residue loop, a site that is not conducive to base stacking and is expected to leave the nucleobase predominantly solvent exposed.^35,36^ ODN4 and ODN7 were selected to provide a first look at neighboring base effects on ABN's fluorescence. By annealing these ODNs to matched and mismatched complementary strands, the base pairing of ABN and its effects on fluorescence can be measured.

### Photophysical properties

#### Steady-state measurements

**Fig. 4:**
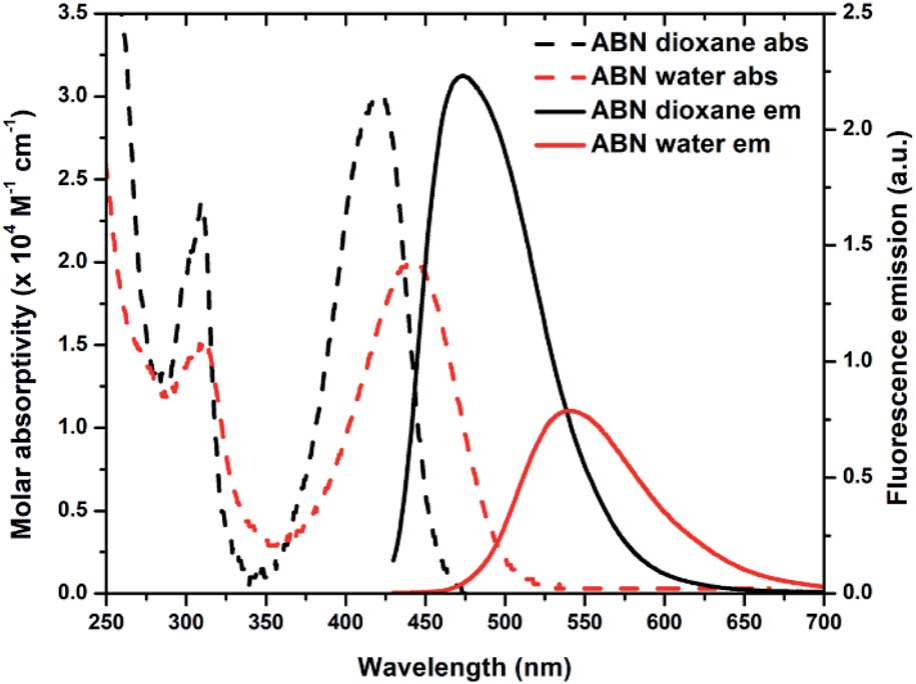
Absorption (dashed line) and emission (solid line) spectra of ABN in dioxane (black) and water (red). The integral areas of emission spectra are normalized to brightness *ε*·*Φ*_em_.

Steady-state absorption and fluorescence measurements of ABN in water, 1× PBS buffer (pH 7.4), 1,4-dioxane, and mixtures indicate that it is among the brightest fluorescent nucleosides reported to date (*ε*_442_ = 20 000 M^−1^ cm^−1^ and *Φ*_em,540_ = 0.39 in water and *ε*_420_ = 30 000 M^−1^ cm^−1^ and *Φ*_em,474_ = 0.64 in 1,4-dioxane; Table 1; Fig. 4 and S19†). Next to its closest competitors on brightness, pentacyclic adenine pA (*ε*_387_ = 15 300 M^−1^ cm^−1^ and *Φ*_em,420_ = 0.66 in water) and a coumarin nucleoside (*ε*_315_ = 38 000 M^−1^ cm^−1^ and *Φ*_em,455_ = 0.11 in water), the absorption and emission of ABN in aqueous solution are red-shifted by more than 50 and 80 nm, respectively.^16,17^ Owing to the conjugated push–pull system in the design of ABN, the absorption and emission wavelengths are the longest known for a FBA designed to be capable of Watson–Crick hydrogen bonding. Recorded emission spectra in water using excitation wavelengths ranging from 310–500 nm are nearly superposable, as are excitation spectra recorded for emission wavelengths spanning 500–650 nm (Fig. S20 and S21†). These observations support the predominance of a single tautomeric form in dilute, aqueous solution (Fig. S20†).

**Table 1:** Steady-state photophysical data for the ABN nucleoside

Solvent	*λ*_ex, max_/nm	*λ*_em, max_/nm	*ε* at *λ*_ex, max_/M^−1^ cm^−1^	*Φ*_em_	*ε*·*Φ*_em_
H_2_O	442	540	2.0 × 10^4^	0.39	7800
1× PBS pH 7.4	442	540	n.d.^a^	0.39	—
1,4-Dioxane	420	474	3.0 × 10^4^	0.64	19 000

a n.d. = not determined.

Next, we measured the fluorescence of ABN in single-stranded and duplex DNA oligonucleotides to determine how base pairing and stacking influence its photophysical properties (Table 2). ABN increases its fluorescence to *Φ*_em_ = 0.50–0.53 in matched duplex DNA oligonucleotides when base paired with A and *Φ*_em_ = 0.55 when the analogue is present in the hairpin loop of ODN1. The quantum yield is less, 0.40 and 0.29, in ODN1:ODN3 and ODN4:ODN6 respectively, in which ABN is base paired with G. In ODN7:ODN9 *Φ*_em_ = 0.55, slightly greater than when base paired with A. In all of these single-stranded and duplex sequences, ABN is brighter than any other known FBA when present in oligonucleotides.

**Table 2:** Steady-state photophysical data for ABN in DNA oligonucleotides

Oligo^a^	*λ*_abs, max_/nm	*λ*_em, max_/nm	*Φ*_em_	*T*_m_/°C	Δ*T*_m_^b^/°C
ODN1	450	530	0.55	63.6 ± 0.6	+3.1
ODN1:ODN2	440	530	0.53	61.8 ± 0.4	−4.7
ODN1:ODN3	468	523	0.40	61.3 ± 0.6	+1.6^c^
ODN4	452	540	0.49	—	—
ODN4:ODN5	440	525	0.51	39.9 ± 0.3	−0.5
ODN4:ODN6	470	523	0.29	41.3 ± 0.3	−7.3^c^
ODN7	d	532	0.62	—	—
ODN7:ODN8	d	532	0.50	34.3 ± 0.3	−14.1
ODN7:ODN9	d	532	0.55	37.9 ± 0.3	−11.8^c^

a Oligonucleotide sequences are given in Fig. 3.

b Δ*T*_m_ = *T*_m_ for ABN-containing duplex listed in the table row − *T*_m_ for the corresponding duplex with canonical thymidine in place of ABN.

c Comparison with *T*_m_ for the corresponding natural duplex with a C:G base pair.

d *λ*_ex,max_ is concentration-dependent; see Fig. S18.

Temperature-dependent circular dichroism measurements of all six duplexes are consistent with the B-DNA conformation with only minimal perturbation (Table 2; see all CD spectra in the ESI†). Except in the ODN1 hairpin, where ABN is expected to be mostly solvent exposed, the melting temperatures of the duplexes are typically somewhat depressed as compared with their natural counterparts. Duplex stability is lowest when ABN has 5′-G and 3′-C neighbors, an observation consistent with other tricyclic FBAs, especially those that are electron-rich.^14^ The observed melting temperatures provide little indication of whether ABN is a better T- or C-surrogate. Solution NMR studies of the free nucleoside and computation clearly indicate a preferred tautomer with an acceptor–donor–acceptor hydrogen bonding pattern as in thymine, as discussed above. It is possible that ABN forms wobble base pairs with G or base pairing with G drives tautomerism to a C-like donor–acceptor–acceptor hydrogen bonding pattern (Fig. S22†).

The Stokes shift is shortened in ABN:G pairs, resulting mostly from red-shifted absorption relative to what is observed in ABN:A pairs. Computational studies (B3LYP/cc-pVDZ) predict that the C-like tautomer will absorb at approximately 45 nm longer wavelength than the T-like tautomer (see ESI†). These calculations are consistent with a tautomeric base pair with G that retains high fluorescence but is indicated by changed *λ*_ex_. The absorption spectra of ODN7 alone and in the ODN7:ODN8 and ODN8:ODN9 duplexes are concentration dependent (Fig. S18†). This dependency indicates a significant potential for change in the local environment around ABN and possibly the analogue's tautomeric state in these sequence contexts.

### Single-molecule fluorescence measurements

**Fig. 5:**
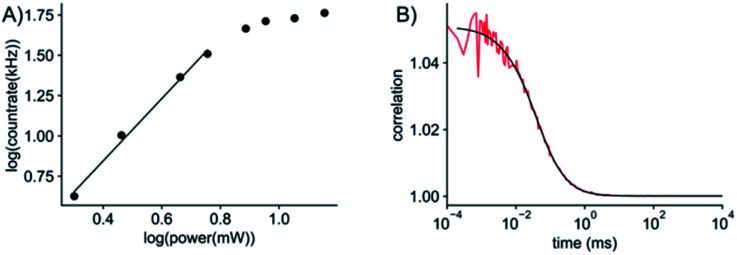
2P single-molecule characterization of ABN. (A) Logarithmic plot of the power dependence of the emission intensity. The first four points show a linear behavior with a slope of 1.9; the slope changes for powers higher than 5.7 mW, presumably due to saturation effects. (B) FCS measurement (red line) and fit to the data (black line). The fit gives an average of 7 molecules in the focus which leads to an average count rate per molecule of 7 ± 0.5 kHz per molecule. The excitation power for FCS was 11 mW (see ESI for details†). All measurements were performed with a 100 nM ABN solution in TRIS buffer.

Given the very attractive bulk-level photophysical properties of ABN in solution, we next investigated its potential as a single-molecule probe. Recent studies have demonstrated multiphoton excitation as a promising approach to the sensitive detection of fluorescent base analogues.^19,20^ Two-photon excitation of pA in oligonucleotides allowed detection close to the single-molecule level, whereas one-photon (1P) excitation resulted in rapid photobleaching.^19^ In a later study, the base analogue DMA^th^aU was detected as a free nucleoside at the single-molecule level for the first time *via* multiphoton excitation with a brightness of ∼7 kHz per molecule following three-photon excitation.^20^ Using an experimental setup consisting of a broadband ultrafast laser with dispersion compensation,^19,20^ we found that ABN could be optimally excited *via* a 2P process (Fig. 5A). The 2P brightness was measured using fluorescence correlation spectroscopy (FCS) and found to match that of DMA^th^aU at 7 kHz per molecule (Fig. 5B). Importantly, unlike DMA^th^aU, which was predominately (96%) in a dark state, a controlled dilution suggests that ABN is exclusively in a bright state, which we attribute to the single tautomer observed by NMR (see above).

**Fig. 6:**
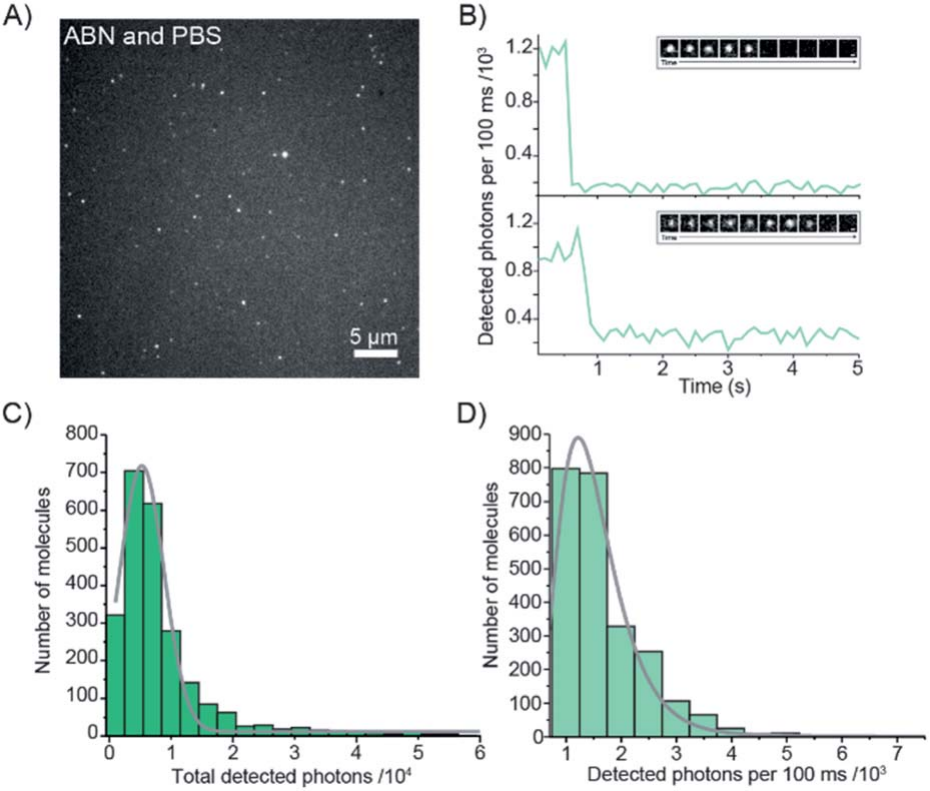
1P single-molecule characterization of ABN. (A) Average fluorescence intensity projection of a 15 s movie of single ABN molecules adsorbed onto a glass surface. (B) Fluorescence intensity as a function of time of two single ABN molecules, with subselections of the traces shown as time montages (inset, scale bar = 200 nm). (C) A histogram showing the distribution of the total number of photons detected from single ABN emitters, *μ* = 5300 ± 1800 photons determined from a log-normal distribution fit. (D) A histogram showing the mean number of photons detected/frame for single ABN emitters, *μ* = 1500 ± 600 photons/100 ms, determined from a log-normal distribution fit. (*N* = 2402 molecules).

The red-shifted absorption profile, high 2P brightness and predominance of the bright state made ABN a promising candidate for single-molecule detection *via* 1P excitation. We found that spatially isolated individual molecules of ABN randomly dispersed on a glass coverslip could be readily visualized using single-molecule total internal reflection fluorescence (smTIRF) microscopy (Fig. 6A and Movie S1†). Furthermore, these fluorescent puncta underwent single-step photobleaching under constant irradiation (Fig. 6B), consistent with the idea that ABN has suitable optical properties to be used as a single-molecule fluorescent nucleoside. By quantifying >2000 individual trajectories, we were able to determine the mean total detected photon value of 5300 ± 1800 photons per molecule; furthermore a mean of 1500 ± 600 photons were detected per 100 ms integration from each molecule at a power density of 0.2 kW cm^−2^. Combined, these data suggest that the mean total on-time of ABN was 0.35 ± 0.18 s under these conditions. The similarities in brightness following 1P and 2P excitation (15 kHz and 7 kHz per molecule, respectively), show that ABN has excellent photostability under both excitation regimes.

## Conclusions

The design of this new fluorescent nucleoside analogue ABN, centered around a push–pull motif common in bright organic fluorophores, has provided an unprecedented combination of high brightness and long absorption and emission wavelengths while retaining a Watson–Crick face. Fluorescence is further enhanced when the compound is present in single-stranded and duplex oligonucleotides. ABN's robust photophysical properties and tautomeric stability allow detection of single molecules of the ABN nucleoside using either 1P or 2P at convenient excitation wavelengths for both. These results place ABN as the most promising fluorescent nucleoside analogue to date for single-molecule studies of nucleic acid structure and dynamics. A forthcoming full study will elucidate the finer details of this analogue's fluorescent properties, base pairing, tautomerism, and local structural perturbations in a variety of neighboring base sequences.

## Conflicts of interest

There are no conflicts to declare.

## Supplementary Material

SC-012-D0SC03903A-s001

SC-012-D0SC03903A-s002
