# Supplementary material for: Single-molecule fluorescence detection of a tricyclic nucleoside analogue
Source: Chem Sci. 2020 Dec 28;12(7):2623–8. doi: 10.1039/d0sc03903a (PMC8179283; doi:10.1039/d0sc03903a)
Supplement: SC-012-D0SC03903A-s001 [file SC-012-D0SC03903A-s001.pdf]

Electronic Supplementary Material (ESI) for Chemical Science.
This journal is © The Royal Society of Chemistry 2021

Single-molecule fluorescence detection of a tricyclic nucleoside analogue

George N. Samaan,[1] Mckenzie K. Wyllie,[1] Julian M. Cizmic,[1] Lisa-Maria Needham,[2] David Nobis,[3] Katrina Ngo,[1] Susan Andersen,[1] Steven W. Magennis,[3] Steven F. Lee,[2] and Byron W. Purse[1],*

(1) Department of Chemistry and Biochemistry and the Viral Information Institute, San Diego State University, San Diego, CA, 92182, USA, (2) University of Cambridge, Chemistry Department, Lensfield Road, Cambridge, CB2 1EW, UK, (3) School of Chemistry, University of Glasgow, University Avenue, Glasgow, G12 8QQ, UK

* bpurse@sdsu.edu

Table of Contents

General experimental 2
Synthesis of ABN 3
NMR spectra 9
Synthesis of oligonucleotides 21
Photophysical measurements 22
- Quantum yields measurements 22
- Circular dichroism spectroscopy 22
- Additional figures 27
- 1P single-molecule methods 30
- 2P single-molecule methods 31

Computational studies 32
References 36

General experimental

Starting materials were obtained in ACS reagent grade or higher from Acros Organics, Alfa Aesar, Fisher Scientific, Sigma-Aldrich and Wako Chemicals and used without further purification. Analytical thin-layer chromatography was performed on pre-coated 200 µm silica gel F-254 plates. Visualization was performed by ultraviolet light. Flash column chromatography was performed using a Teledyne-Isco CombiFlash RF 200 using UV/vis detection. ^{1}H NMR spectra were recorded on 400 and 500 MHz Varian spectrometers using an AutoX PFG probe at 298 K; residual solvent peaks were used as internal references: DMSO (quint, δH = 2.50 ppm), $CHCl_3$ (s, δH = 7.26 ppm) or methanol (quint, δH = 3.31 ppm). ^{13}C NMR spectra were recorded on 400 and 500 MHz Varian spectrometers using an AutoX PFG probe at 298 K; δ relative to DMSO (δ 40.50 ppm), $CHCl_3$ (δ 77.23 ppm) or methanol (δ 49.00 ppm). Coupling constants (*J*) are reported in hertz (Hz). The following abbreviations are used to describe the multiplicities: s = singlet, d = doublet, t = triplet, q = quartet, quint = quintet, sext = sextet, m = multiplet, dd = doublet-doublet, ddd = doublet-doublet-doublet, dt = doublet-triplet, dq =doublet-quartet. High-resolution electrospray ionization (ESI) mass spectrometry was performed using an Agilent 6530 Accurate-Mass Q-TOF LC/MS.

Synthesis of ABN

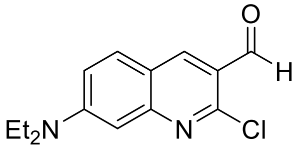

2

2-chloro-7-(diethylamino)quinoline-3-carbaldehyde (2).

$POCl_3$ (27.1 mL, 291 mmol) is placed into a dry flask under nitrogen and cooled in an ice bath, and to this, DMF (5.60 mL, 72.7 mmol) is added portionwise while stirring. 3-(diethylamino)acetanilide (5.00 g, 24.2 mmol) in 1,4-dioxane (7.00 mL) is added to the mixture, and the reaction is heated at 50 °C. After 20 min, the reaction mixture is quenched by transferring it portionwise to a solution of cold saturated $NaHCO_3$. NaOH pellets and ice are added to the bath with stirring until the mixture is alkaline (pH 9). The slurry is filtered and the solid is washed with water then dissolved in DCM and dried over Na_2SO_4 and evaporated to dryness to collect the crude product. These crude products are purified by automated flash chromatography (0–100% EtOAc in hexane) and the product elutes with 30% EtOAc in hexane (R_f = 0.652). The solid is recrystallized using DCM and hexane to give 956 mg (15.0% yield) of yellow solid (**2**).[1]
^{1}H NMR (400 MHz, $CDCl_3$) δ 10.30 (s, 1H), 8.35 (s, 1H), 7.61 (d, *J* = 9.2 Hz, 1H), 7.02 (dd, *J* = 9.2, 2.6 Hz, 1H), 6.90 (d, *J* = 2.5 Hz, 1H), 3.45 (q, *J* = 7.1 Hz, 4H), 1.21 (t, *J* = 7.1 Hz, 6H). **^{13}C NMR** (125 MHz, $CDCl_3$) δ 188.8, 152.1, 151.8, 151.4, 138.8, 131.2, 121.7, 118.4, 116.5, 104.4, 45.0, 12.5. **HR-ESI MS (*m/z*): $[M+H]^+$** calculated for $C_{14}H_{16}ClN_2O$ 263.0951, found 263.0963.

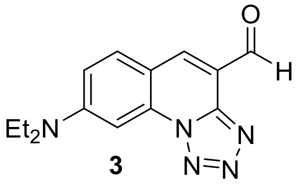

3

8-(diethylamino)tetrazolo[1,5-*a*]quinoline-4-carbaldehyde (3).

(**2**) (3.00 g, 11.5 mmol) and sodium azide (2.23 gm, 34.4 mmol) are dissolved in 7.00 mL of DMF, then the reaction is heated and stirred at 90.0 °C. The reaction is monitored by TLC (70% EtOAc in hexane; R_f = 0.642) and stopped after 18 hours. The reaction mixture is then added to iced water to induce precipitation. The solid is dissolved in 3.00 mL of DCM and induced to crystallize by the addition of 100 mL cold hexane. The solid is collected to give 2.64 g (85.0% yield) of yellow solid (**3**).
^{1}H NMR (500 MHz, $CDCl_3$) δ 10.50 (s, 1H), 8.31 (s, 1H), 7.78 (d, *J* = 9.2 Hz, 1H), 7.57 (d, *J* = 2.5 Hz, 1H), 7.00 (dd, *J* = 9.2, 2.6 Hz, 1H), 3.59 (q, *J* = 7.2 Hz, 4H), 1.32 (t, *J* = 7.2 Hz, 6H). **^{13}C NMR** (125 MHz, $CDCl_3$) δ 186.2, 152.1, 146.9, 136.0, 135.0, 132.9, 114.7, 114.3, 112.4, 95.5, 45.4, 12.5. **HR-ESI MS (*m/z*): $[M+H]^+$** calculated for $C_{14}H_{16}N_5O$ 270.1355, found 270.1365.

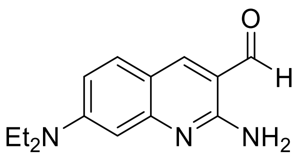

4

2-amino-7-(diethylamino)quinoline-3-carbaldehyde (4).

(**3**) (1.00 gm, 3.72 mmol) and triphenylphosphine (1.95 gm, 7.44 mmol) are added to a round-bottom flask. 5.00 mL of methanol and 18.0 mL of 2.00 N aqueous HCl are added and the reaction is heated at reflux, monitoring by TLC (5% methanol in DCM; R_f = 0.320). After 2 hours, the reaction is cooled to room temperature, ice water (200 mL) is added, and the mixture is filtered. 2.00 N NaOH is added portion-wise to the filtrate until yellow solid precipitates. The slurry is filtered and the yellow solid is collected to give 633 mg (70.0% yield) of the product (**4**).

^{1}H NMR (400 MHz, $CDCl_3$) δ 9.79 (s, 1H), 8.00 (s, 1H), 7.47 (d, *J* = 9.1 Hz, 1H), 6.78 (dd, *J* = 9.1, 2.5 Hz, 1H), 6.61 (d, *J* = 2.5 Hz, 1H), 3.47 (q, *J* = 7.1 Hz, 4H), 1.23 (t, *J* = 7.1 Hz, 6H). **^{13}C NMR** (101 MHz, $CDCl_3$) δ 191.4, 156.5, 152.8, 152.3, 146.9, 130.9, 115.0, 113.4, 112.1, 102.2, 44.9, 12.9. **HR-ESI MS (*m/z):* [M+H]$^+$** calculated for $C_{14}H_{18}N_3O$ 244.1450, found 244.1461.

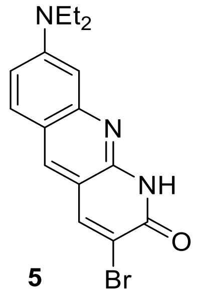

5

3-bromo-8-(diethylamino)benzo[*b*][1,8]naphthyridin-2(1*H*)-one (5).

(**4**) (671 mg, 2.75 mmol) and (1.76 g, 4.13 mmol) ethyl 2-bromo-2-(triphenylphosphoranylidene)acetate, synthesized by bromination of ethyl (triphenylphosphoranylidene)acetate,[2] (are dissolved in 10.0 mL of ethanol, and 20% sodium ethoxide (1.87 mL, 5.51 mmol) is added and the reaction is heated at 70.0 °C. The reaction is monitored by TLC (5% methanol in DCM; R_f = 0.519) and after 4 h, is cooled to room temperature. The solvent is evaporated under vacuum then automated flash chromatography is performed (0–100%EtOAc in DCM) and the product coelutes with triphenylphosphine oxide at 20% EtOAc in DCM. This crude product mixture is used directly in the next step.[3]

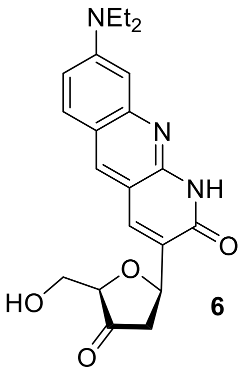

6

8-(diethylamino)-3-((2*R*,5*R*)-5-(hydroxymethyl)-4-oxotetrahydrofuran-2-yl)benzo[*b*][1,8]naphthyridin-2(1*H*)-one (6).

Palladium acetate (156 mg, 0.696 mmol) and triphenylarsine (426 mg, 1.39 mmol) are dissolved in 7.00 mL DMF and stirred for 30 min at room temperature. The reaction mixture is then transferred to another flask containing the crude product (**5**) (300 mg, 0.870 mmol) and 1,4-anhydro-3,5-bis-O-(tert-butyldimethylsilyl)2-deoxy-D-erythro-pent-1-enitol (**10**; see structure in manuscript Figure 2)[4] (599 mg, 1.74 mmol), then tributylamine (413 µL, 1.74 mmol) is added. The reaction is heated to 60.0 °C and monitored by TLC (50% DCM in EtOAc). After 18 h, the reaction is cooled to room temperature then 0.500 mL acetic acid and 1M tetrabutylammonium fluoride (1.74 mL, 1.74 mmol) are added and the reaction is stirred for 1 h at room temperature. The solvent is then evaporated under vacuum and automated flash chromatography is performed (0–100% EtOAc in hexane), with semi-pure product eluting at 90% EtOAc in hexane.[5]

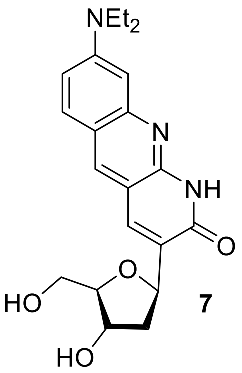

7

8-(diethylamino)-3-((2*R*,4*R*,5*R*)-4-hydroxy-5-(hydroxymethyl)tetrahydrofuran-2-yl)benzo[*b*][1,8]naphthyridin-2(1*H*)-one (7).

The semi-pure product (**6**) and sodium triacetoxyborohydride (277 mg, 1.31 mmol) are dissolved in 3.00 mL acetonitrile and 3.00 mL acetic acid at 0 °C. The reaction is stirred for 2 h at that temperature and monitored by TLC (10% methanol in DCM; R_f = 0.435). The solvent is then evaporated and automated flash chromatography is performed with the product eluting at 10% methanol in DCM. The solvent is evaporated to give 94.8 mg (9.00% yield over the last 4 steps) of yellow solid (**7**).[6]

^{1}H NMR (500 MHz, MeOD) δ 8.29 (s, 1H), 8.07 (d, *J* = 1.1 Hz, 1H), 7.74 (d, *J* = 9.2 Hz, 1H), 7.16 (dd, *J* = 9.3, 2.5 Hz, 1H), 6.88 (d, *J* = 2.4 Hz, 1H), 5.24 (dd, *J* = 10.0, 5.9 Hz, 1H), 4.33 (dt, *J* = 5.5, 2.4 Hz, 1H), 3.99 (td, *J* = 4.6, 2.9 Hz, 1H), 3.73 (qd, *J* = 11.8, 4.6 Hz, 2H), 3.56 (q, *J* = 7.1 Hz, 4H), 2.43 (ddd, *J* = 13.1, 5.9, 2.1 Hz, 1H), 2.00 – 1.93 (m, 1H), 1.26 (t, *J* = 7.1 Hz, 6H). **^{13}C NMR** (125 MHz, MeOD) δ 165.1, 151.9, 151.5, 149.8, 137.6, 136.2, 132.5, 130.9, 119.7, 116.4, 112.9, 103.3, 88.8, 77.3, 74.3, 64.0, 45.7, 42.6, 13.0. **HR-ESI MS (*m/z*): [M+H]$^+$** calculated for $C_{21}H_{26}N_3O_4$ 384.1923, found 384.1945.

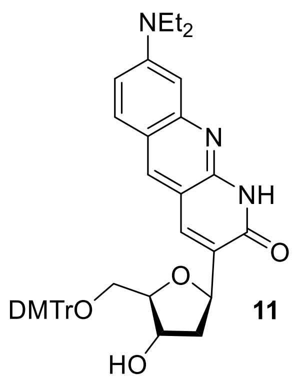

11

3-((2*R*,4*R*,5*R*)-5-((bis(4-methoxyphenyl)(phenyl)methoxy)methyl)-4-hydroxytetrahydrofuran-2-yl)-8-(diethylamino)benzo[*b*][1,8]naphthyridin-2(1*H*)-one (11).
The nucleoside (**7**) (80 mg, 0.209 mmol) and 4,4'-dimethoxytrityl chloride (106 mg, 0.314 mmol) are dried under vacuum for 18 h then 2.5 mL of pyridine is added under nitrogen. The reaction mixture is stirred at room temperature for 1 h. The reaction is monitored by TLC (5% methanol in DCM) and quenched with methanol. The solvent is then evaporated and automated flash chromatography is performed with the product eluting at 3-4% methanol in DCM (containing 1% triethylamine). The solvent from chromatography is evaporated to give 129 mg (90.0% yield) of yellow solid (**11**).
^{1}H NMR (500 MHz, $CDCl_3$) δ 8.93 (s, 1H), 7.98 (d, *J* = 1.3 Hz, 1H), 7.76 (s, 1H), 7.57 (d, *J* = 9.2 Hz, 1H), 7.49 (d, *J* = 7.7 Hz, 2H), 7.41 – 7.35 (m, 4H), 7.28 (t, *J* = 7.8 Hz, 2H), 7.22 (t, *J* = 7.3 Hz, 1H), 7.03 (dd, *J* = 9.3, 2.5 Hz, 1H), 6.90 (d, *J* = 2.4 Hz, 1H), 6.83 (d, *J* = 8.3 Hz, 4H), 5.36 (t, *J* = 7.5 Hz, 1H), 4.43 (s, 1H), 4.09 (d, *J* = 4.2 Hz, 1H), 3.76 (s, 6H), 3.52 (q, *J* = 7.1 Hz, 4H), 3.41 – 3.35 (m, 2H), 2.63 – 2.55 (m, 1H), 2.04 (dt, *J* = 14.0, 6.9 Hz, 1H), 1.27 (t, *J* = 7.0 Hz, 6H). **^{13}C NMR** (125MHz, $CDCl_3$) δ 162.6, 158.7, 150.5, 148.5, 145.0, 136.2, 135.9, 133.8, 132.9, 130.3, 129.7, 128.4, 128.0, 127.0, 118.3, 114.9, 113.4, 111.8, 103.1, 85.4, 77.2, 75.2, 73.9, 64.2, 55.4, 44.9, 41.9, 12.9. **HR-ESI MS (*m/z*): $[M+H]^+$** calculated for $C_{42}H_{44}N_3O_6$ 686.3230, found 686.3229.

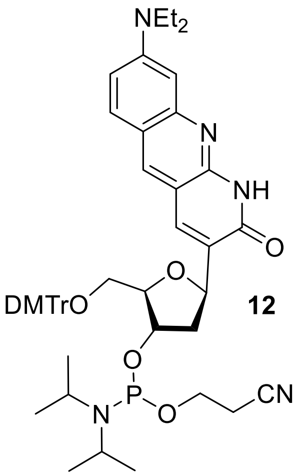

12

(2*R*,3*R*,5*R*)-2-((bis(4-methoxyphenyl)(phenyl)methoxy)methyl)-5-(8-(diethylamino)-2-oxo-1,2-dihydrobenzo[*b*][1,8]naphthyridin-3-yl)tetrahydrofuran-3-yl-2-cyanoethyldiisopropylphosphoramidite (12).

The tritylated nucleoside (**11**) (100 mg, 0.146 mmol) is dissolved in 3 mL DCM then N,N-diisopropylethylamine (102 µL, 0.583 mmol) and 2-cyanoethyl-N,N-diisopropylchlorophosphoramidite (48.9 µL, 0.219 mmol) are added under nitrogen. The reaction mixture is stirred at room temperature for 3 h and monitored by TLC (5% methanol in DCM). The solvent is then evaporated and automated flash chromatography is performed with the product eluting at 0-1% EtOAc in DCM (containing 1% triethylamine). The solvent from chromatography is evaporated, then the product is dissolved in minimal amount of DCM and recrystallized in cold hexane to give 60 mg (46.4% yield) of yellow solid (**12**).

^{1}H NMR (500 MHz, $CDCl_3$) δ 8.84 (s, 1H), 8.07 (s, 0.5H), 8.02 (s, 0.5H), 7.72 (s, 0.5H), 7.65 (s, 0.5H), 7.59 – 7.54 (m, 1H), 7.51 (t, *J* = 7.5 Hz, 2H), 7.39 (td, *J* = 8.7, 2.2 Hz, 4H), 7.28 (d, *J* = 7.6 Hz, 2H), 7.22 (d, *J* = 7.6 Hz, 1H), 7.03 (d, *J* = 9.4 Hz, 1H), 6.89 (s, 1H), 6.83 (t, *J* = 7.6 Hz, 4H), 5.35 (t, *J* = 7.4 Hz, 1H), 4.54 (d, *J* = 30.6 Hz, 1H), 4.24 – 4.18 (m, 1H), 3.87 – 3.81 (m, 1H), 3.76 (s, 6H), 3.65 – 3.59 (m, 2H), 3.52 (q, *J* = 7.1 Hz, 4H), 3.44 – 3.39 (m, 1H), 3.35 – 3.27 (m, 1H), 2.62 (d, *J* = 6.3 Hz, 1H), 2.43 (t, *J* = 6.5 Hz, 1H), 2.06 – 1.94 (m, 1H), 1.27 (t, *J* = 7.0 Hz, 6H), 1.17 (m, 6H), 1.06 (d, *J* = 6.8 Hz, 3H), 0.97 (d, *J* = 6.6 Hz, 1H), 0.91 – 0.84 (m, 4H). **^{31}P NMR** (202 MHz, $CDCl_3$) δ 148.7, 147.9. **HR-ESI MS (*m/z*): [M+H]$^+$** calculated for $C_{51}H_{61}N_5O_7P$ 886.4309, found 886.4310.

NMR spectra

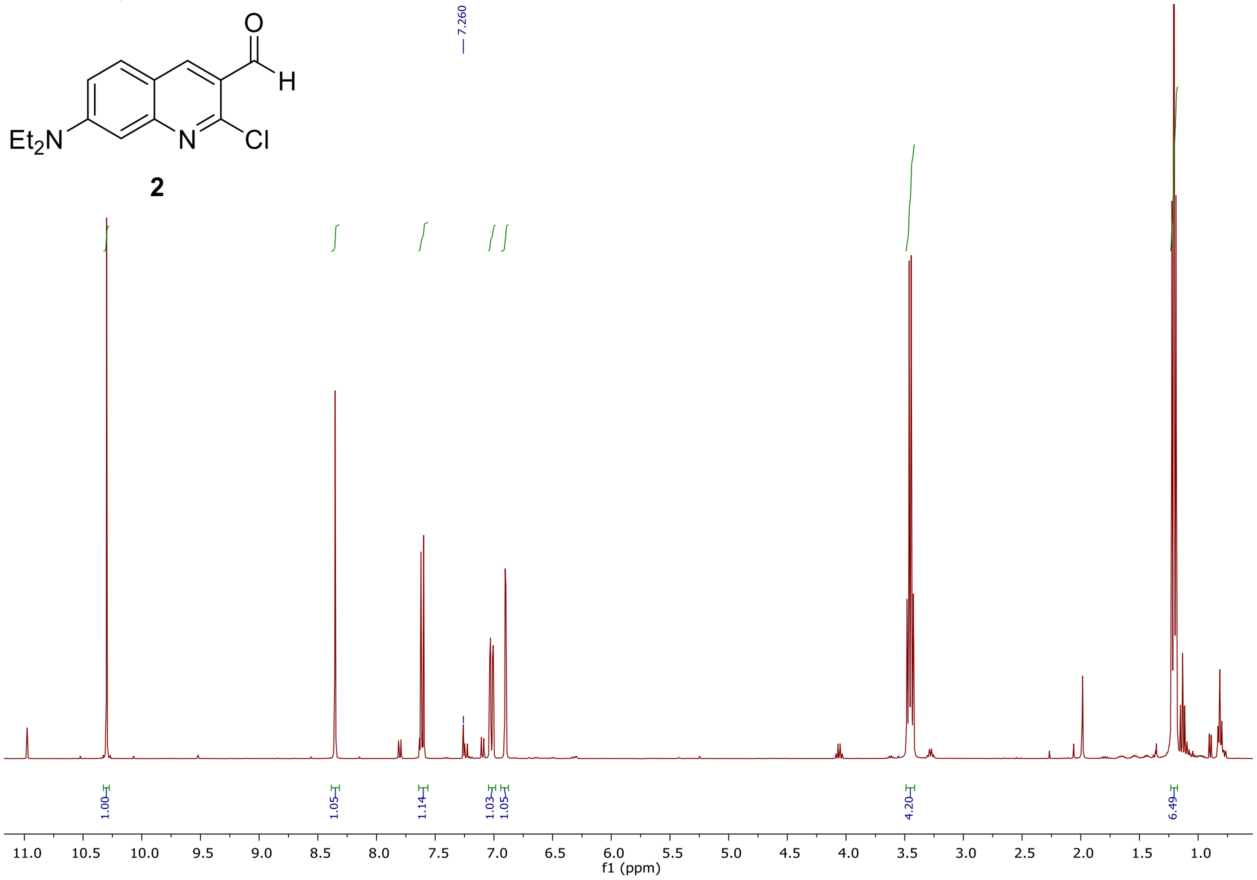

Figure S1. **^{1}H NMR** of 2-chloro-7-(diethylamino)quinoline-3-carbaldehyde **2** (400 MHz; 298 K; $CDCl_3$).

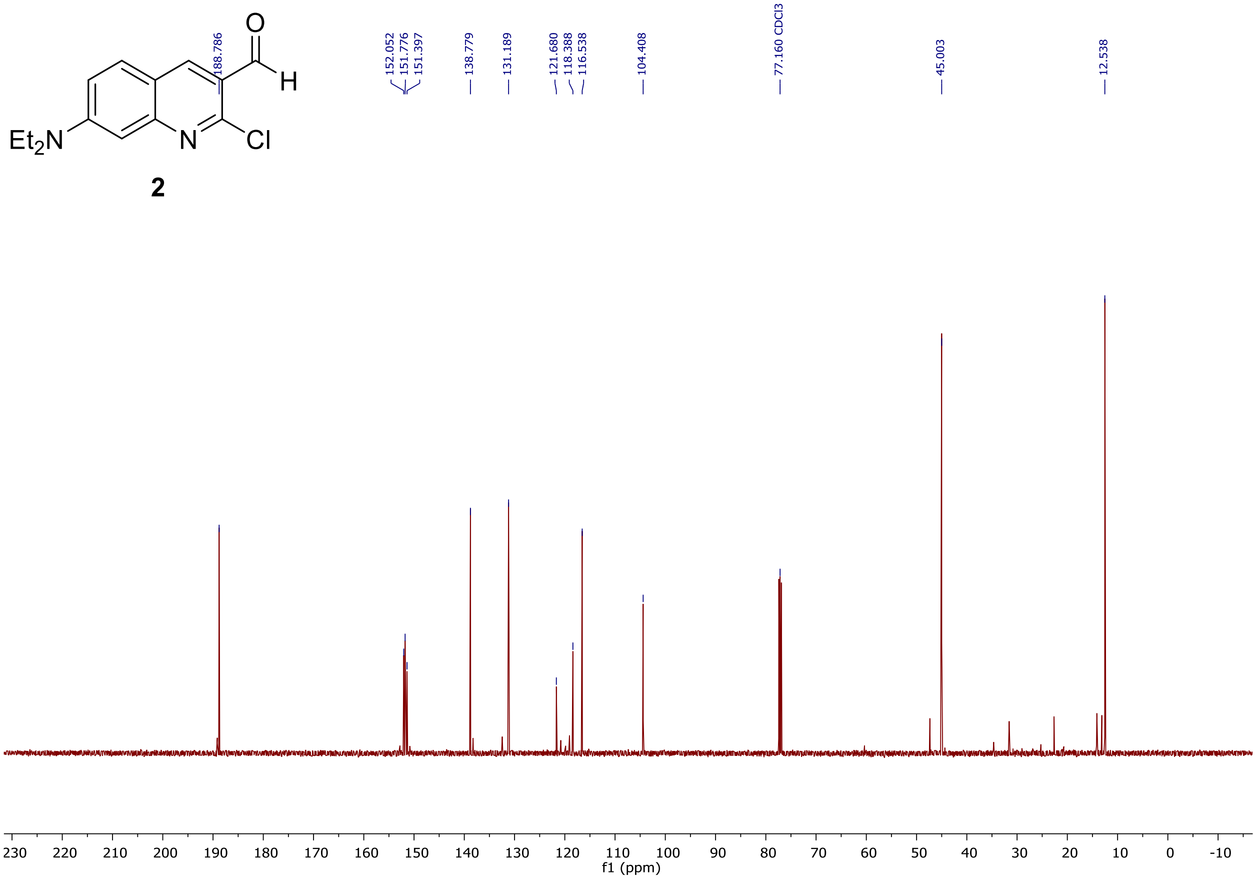

Figure S2. **^{13}C NMR** of 2-chloro-7-(diethylamino)quinoline-3-carbaldehyde **2** (125 MHz; 298 K; $CDCl_3$).

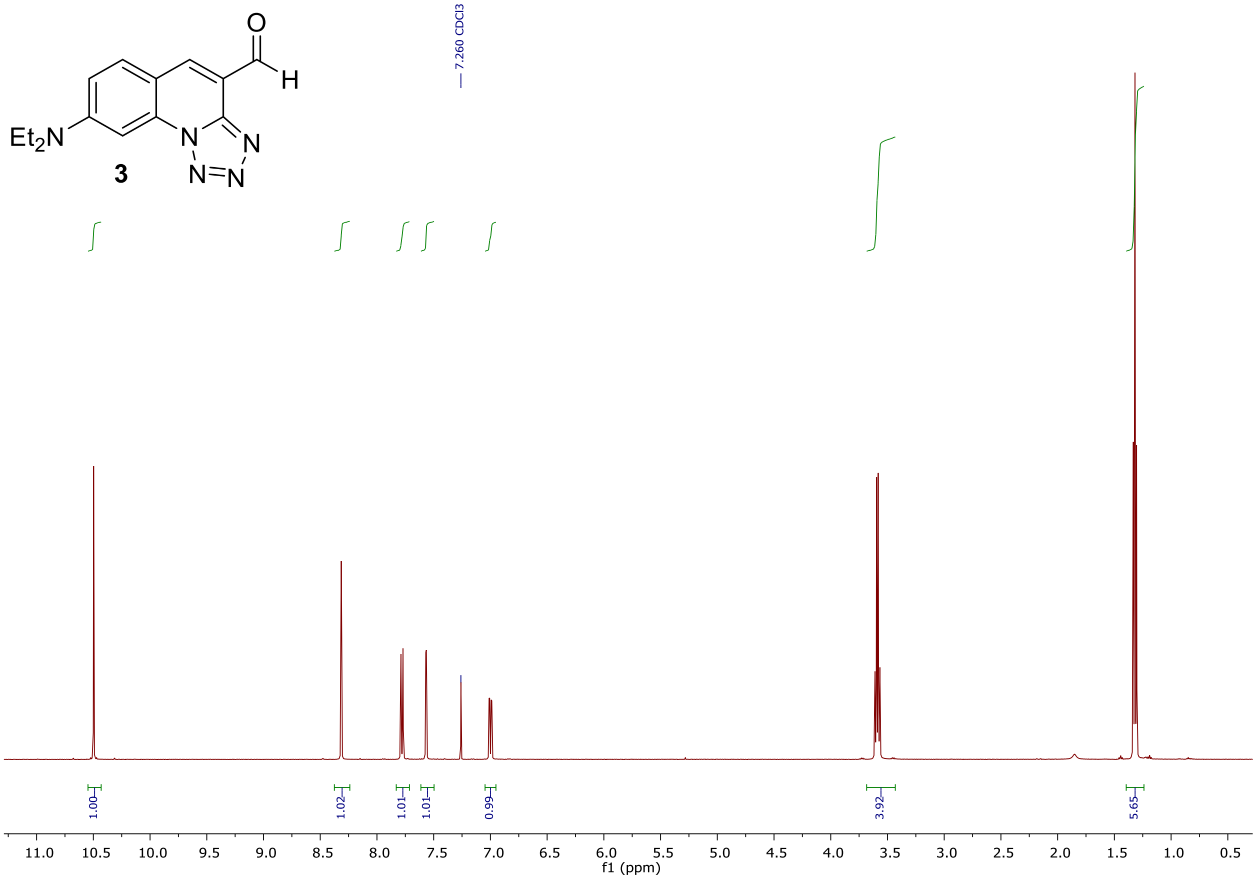

Figure S3.^{1}**H NMR** of 8-(diethylamino)tetrazolo[1,5-*a*]quinoline-4-carbaldehyde **3** (500 MHz; 298 K; $CDCl_3$).

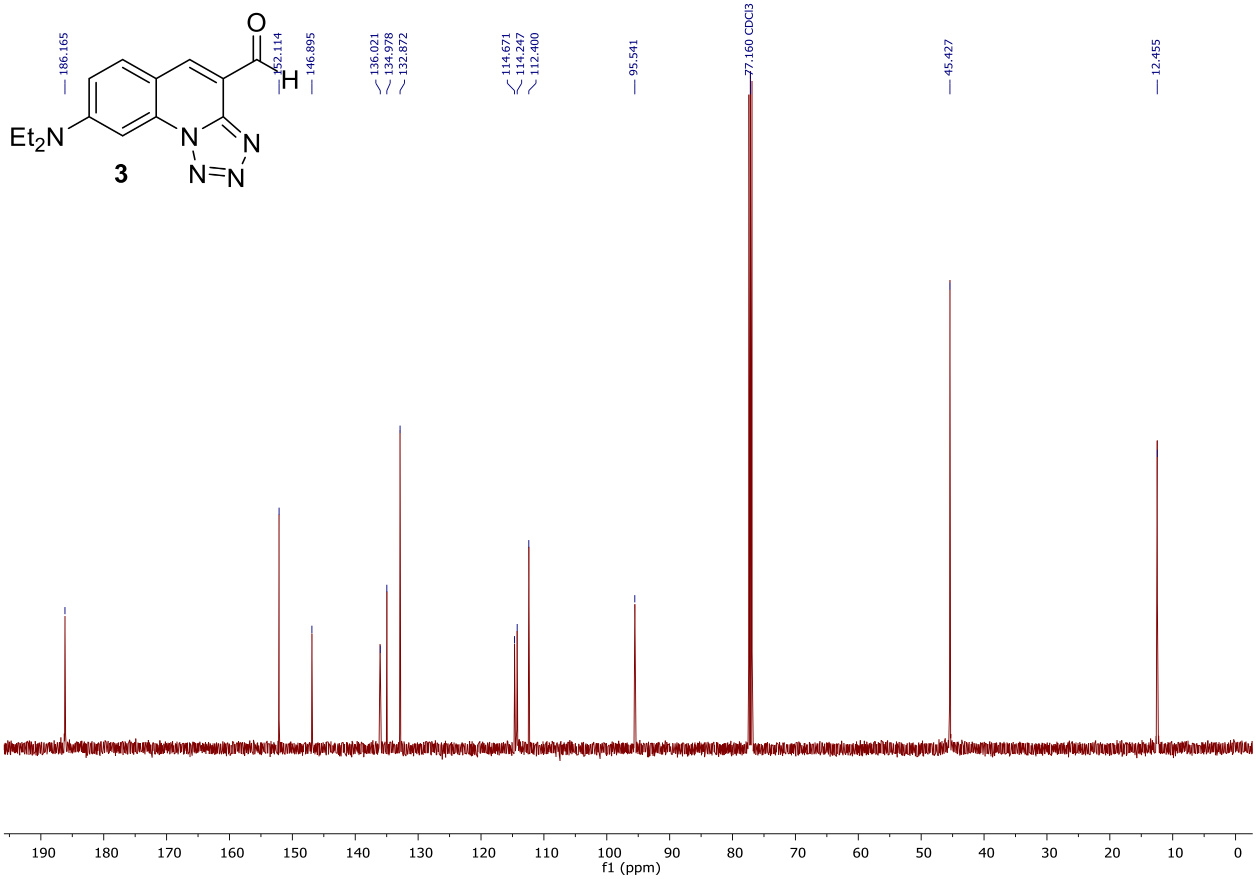

Figure S4. **^{13}C NMR** of 8-(diethylamino)tetrazolo[1,5-*a*]quinoline-4-carbaldehyde **3** (125 MHz; 298 K; $CDCl_3$).

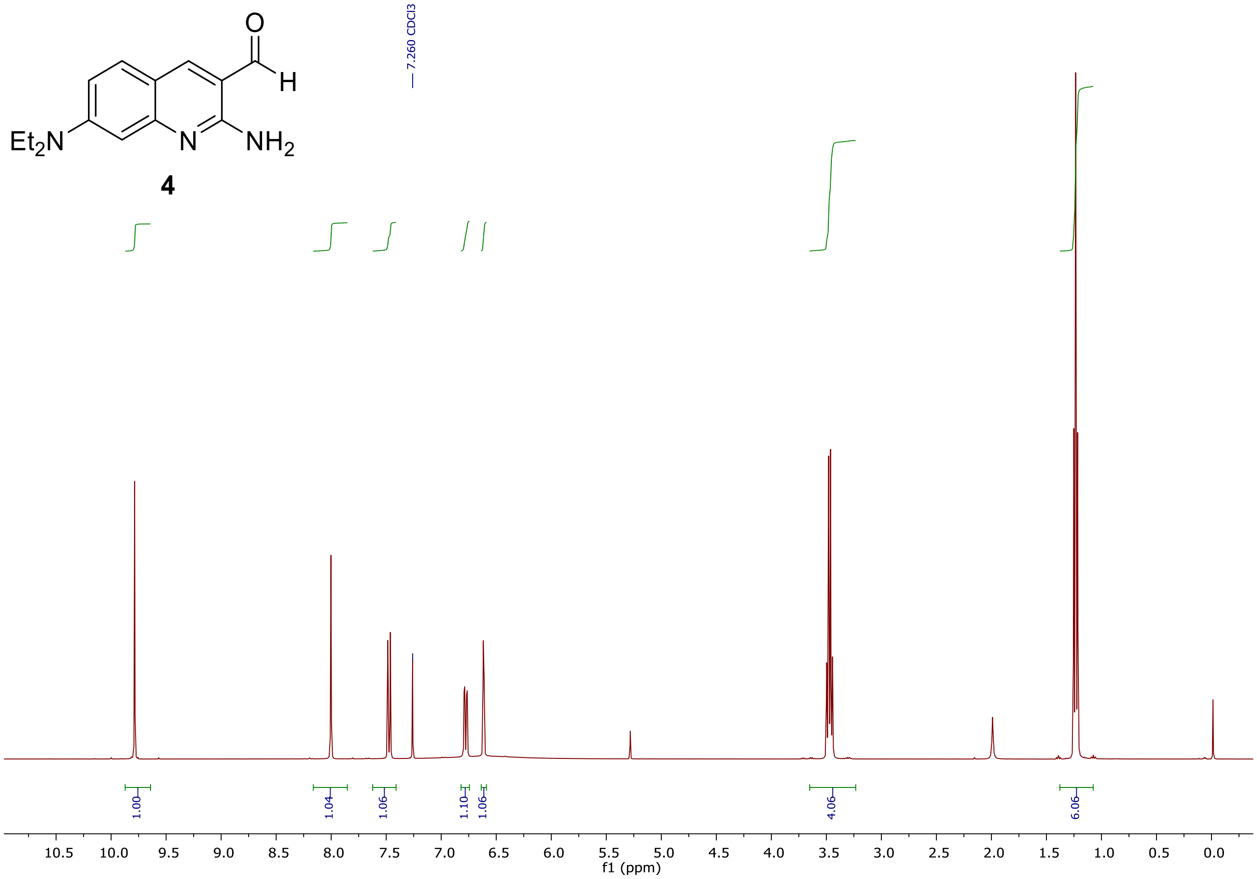

Figure S5.[1]**H NMR** of 2-amino-7-(diethylamino)quinoline-3-carbaldehyde **4** (400 MHz; 298 K; $CDCl_3$).

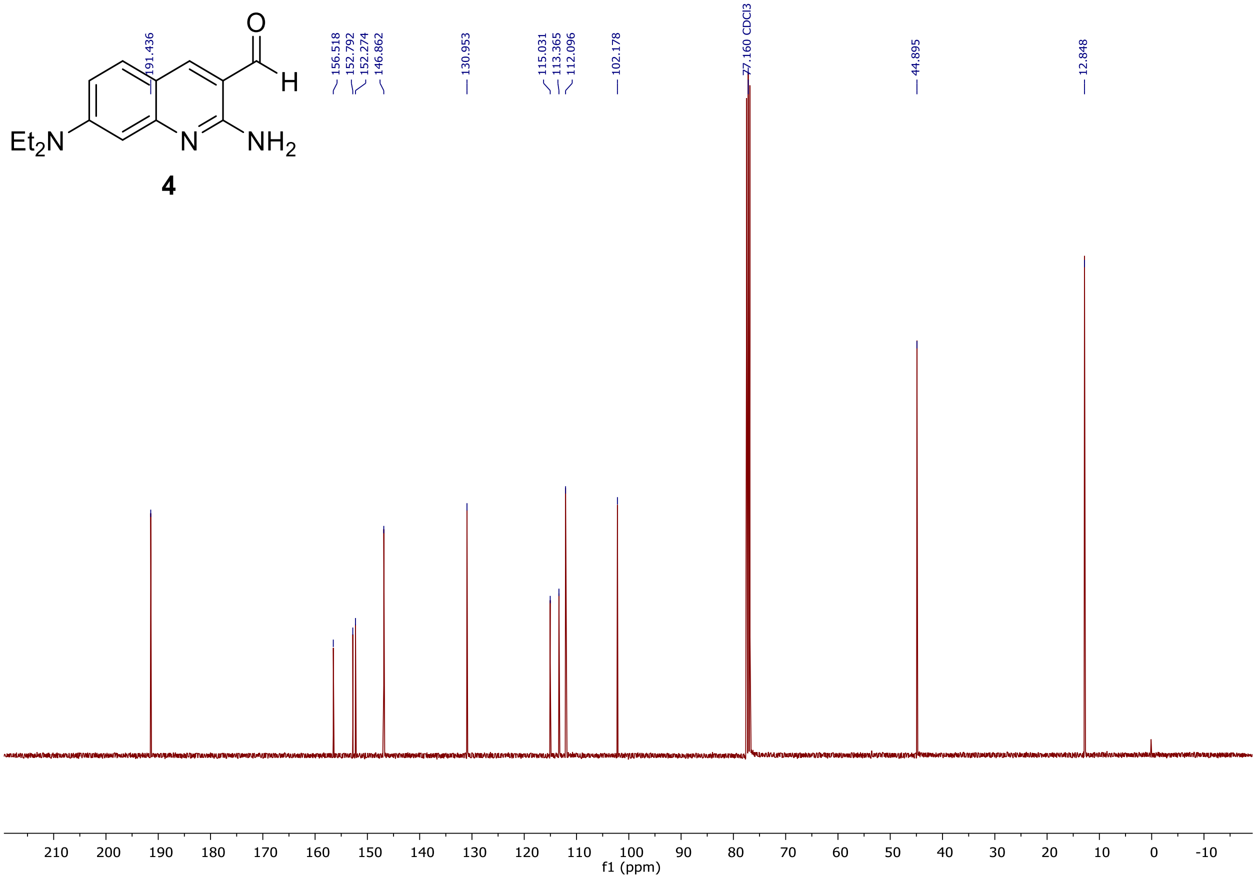

Figure S6. **^{13}C NMR** of 2-amino-7-(diethylamino)quinoline-3-carbaldehyde **4** (101 MHz; 298 K; $CDCl_3$).

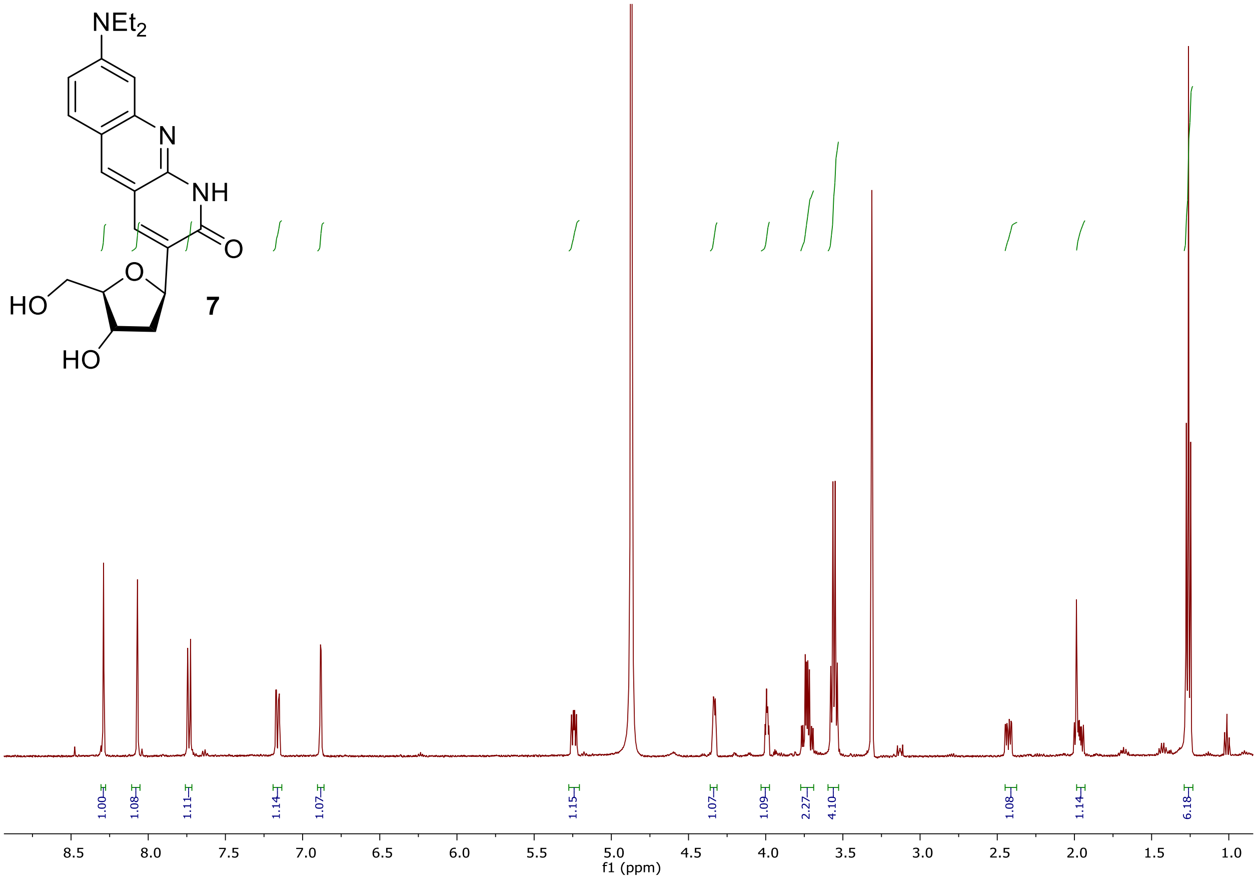

Figure S7.[1]**H NMR** of 8-(diethylamino)-3-((2*R*,4*R*,5*R*)-4-hydroxy-5-(hydroxymethyl)tetrahydrofuran-2-yl)benzo[*b*][1,8]naphthyridin-2(1*H*)-one **7** (500 MHz; 298 K; MeOD).

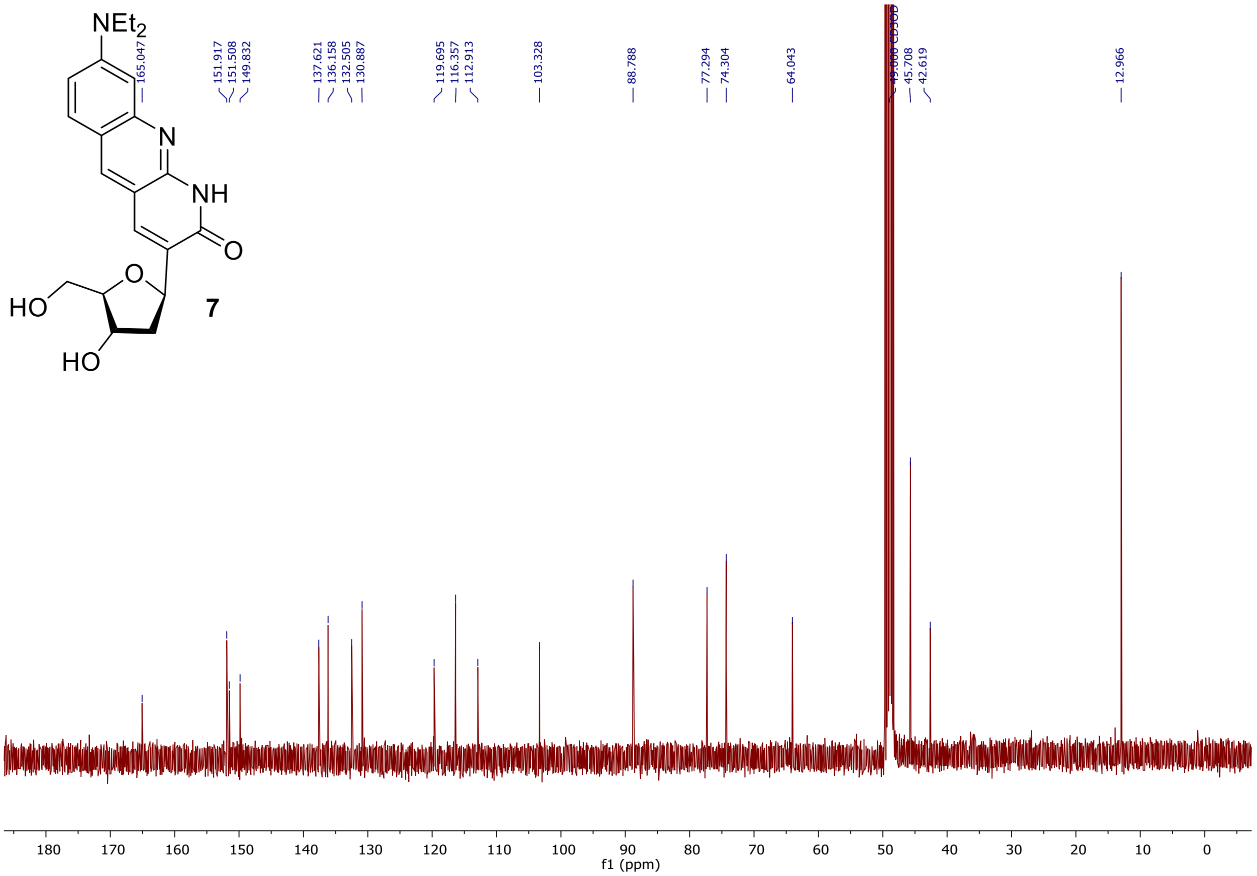

Figure S8.**^{13}C NMR** of 8-(diethylamino)-3-((2*R*,4*R*,5*R*)-4-hydroxy-5-(hydroxymethyl)tetrahydrofuran-2-yl)benzo[*b*][1,8]naphthyridin-2(1*H*)-one **7** (125 MHz; 298 K; MeOD).

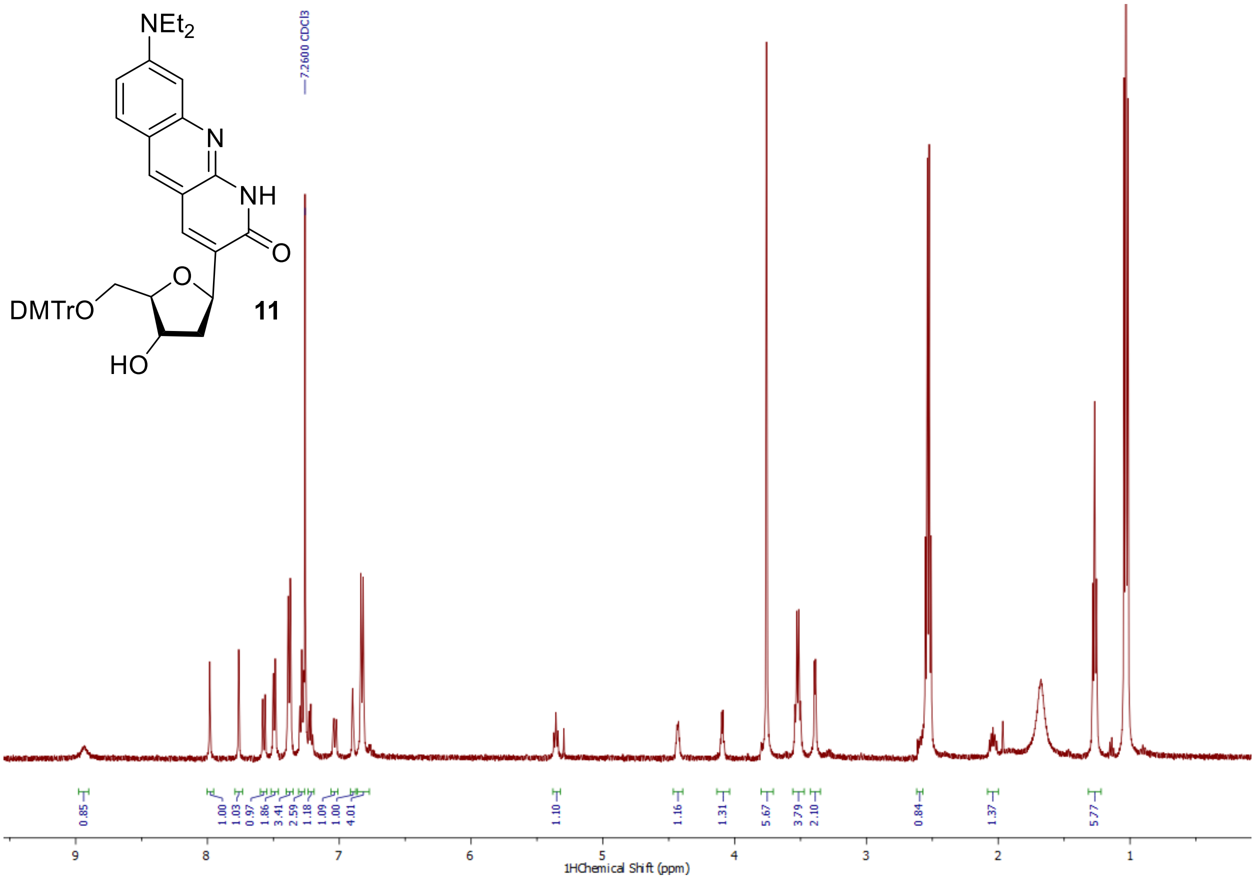

Figure S9. ^{1}**H NMR** of 3-((2*R*,4*R*,5*R*)-5-((bis(4-methoxyphenyl)(phenyl)methoxy)methyl)-4-hydroxytetrahydrofuran-2-yl)-8-(diethylamino)benzo[*b*][1,8]naphthyridin-2(1*H*)-one **11** (500 MHz; 298 K; $CDCl_3$).

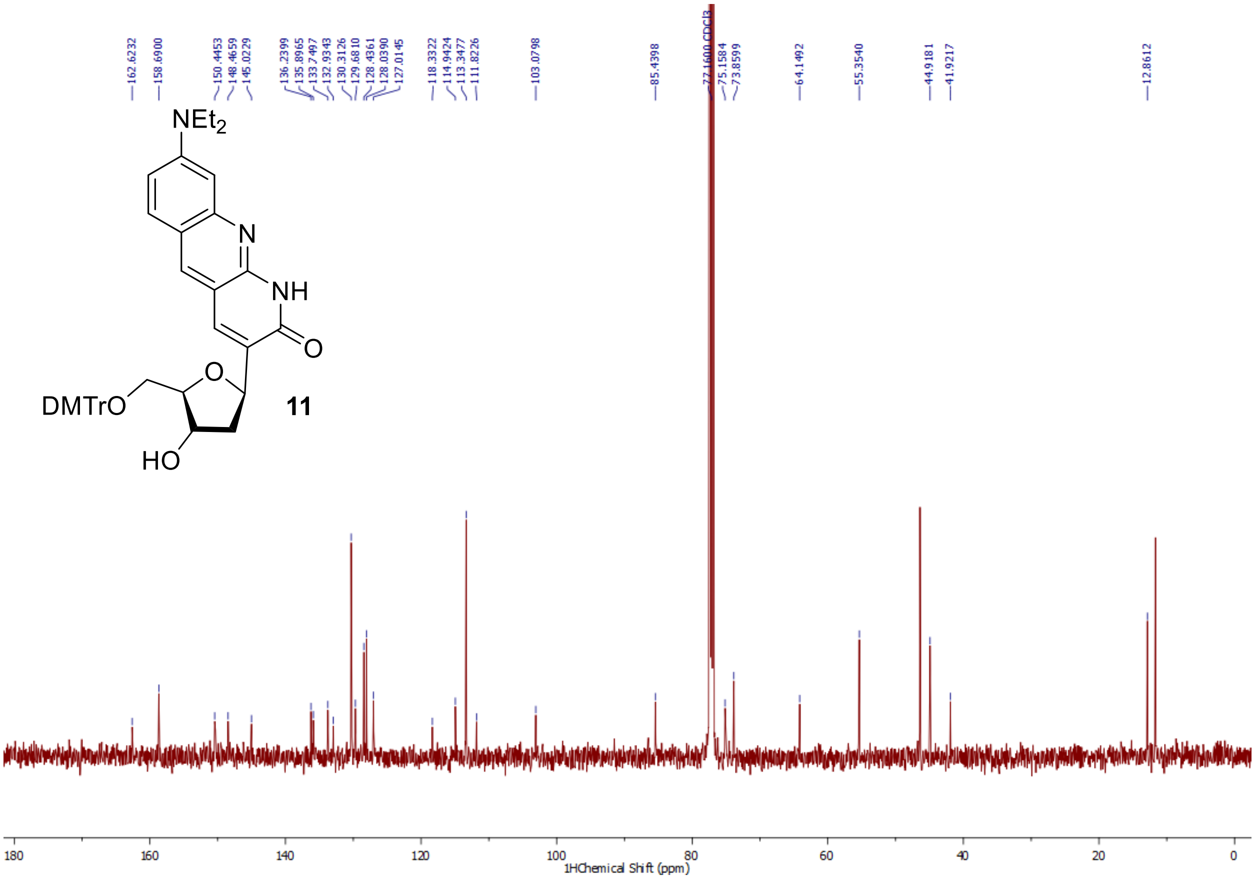

Figure S10.[13]**C NMR** of 3-((2*R*,4*R*,5*R*)-5-((bis(4-methoxyphenyl)(phenyl)methoxy)methyl)-4-hydroxytetrahydrofuran-2-yl)-8-(diethylamino)benzo[*b*][1,8]naphthyridin-2(1*H*)-one **11** (125 MHz; 298 K; $CDCl_3$).

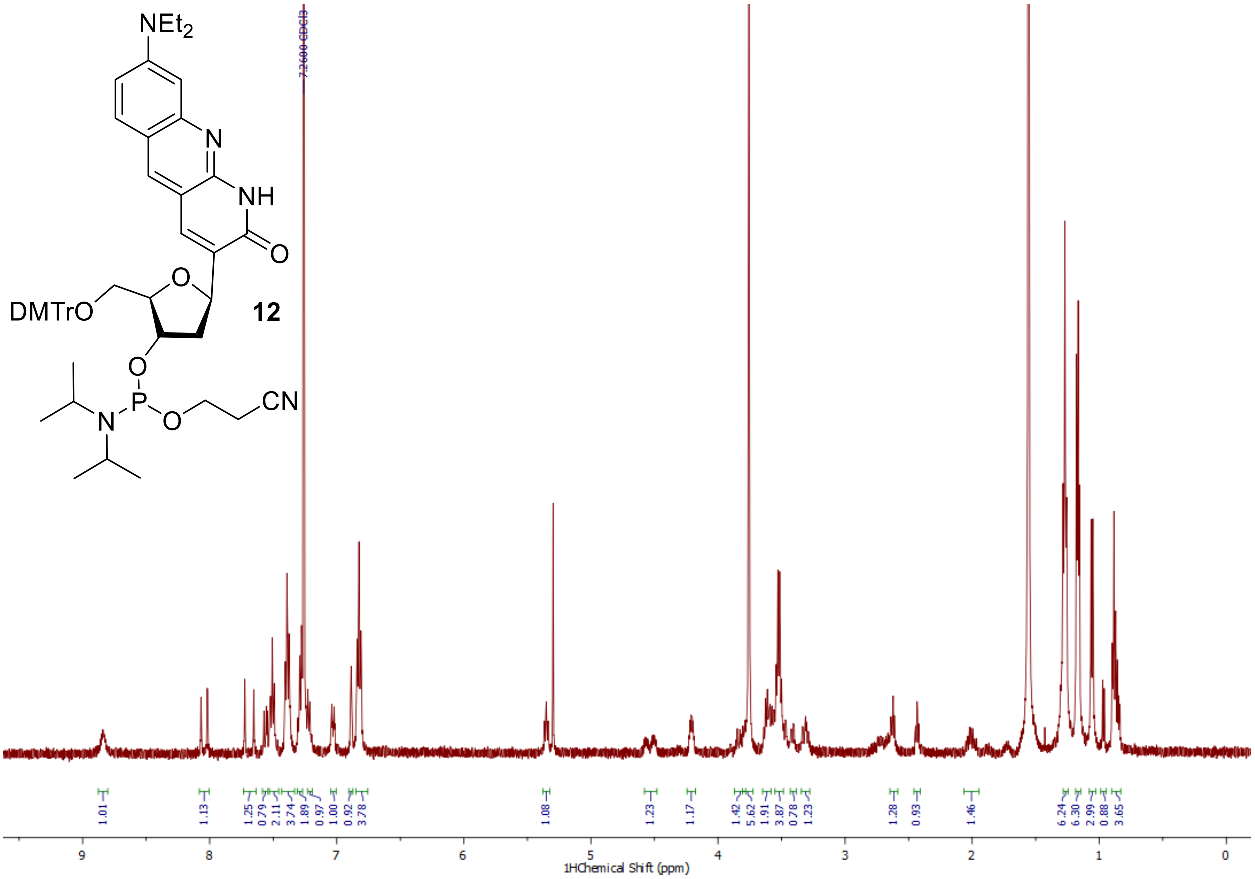

Figure S11.**¹H NMR** of (2*R*,3*R*,5*R*)-2-((bis(4-methoxyphenyl)(phenyl)methoxy)methyl)-5-(8-(diethylamino)-2-oxo-1,2-dihydrobenzo[*b*][1,8]naphthyridin-3-yl)tetrahydrofuran-3-yl-2-cyanoethyldiisopropylphosphoramidite **12** (500 MHz; 298 K; $CDCl_3$).

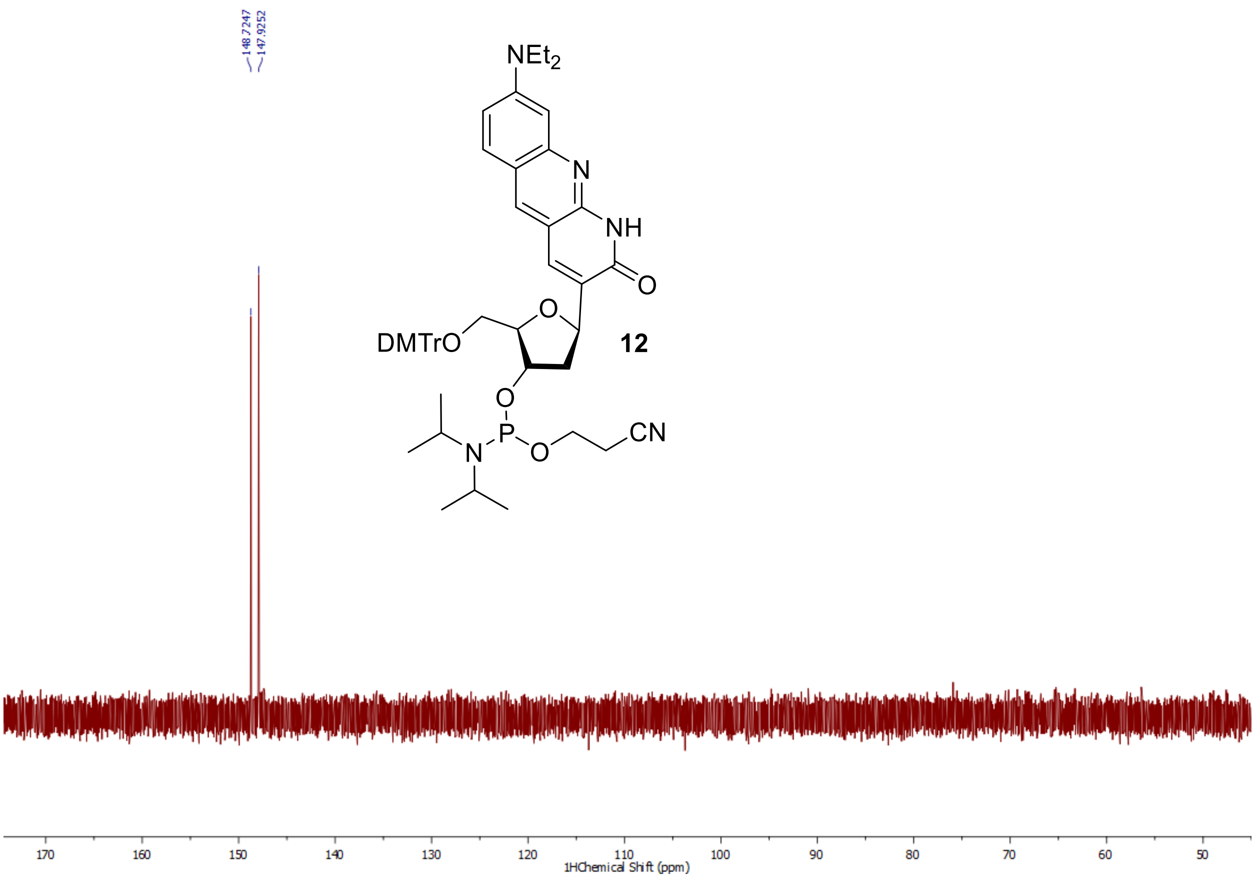

Figure S12. ^{31}P **NMR** of (2*R*,3*R*,5*R*)-2-((bis(4-methoxyphenyl)(phenyl)methoxy)methyl)-5-(8-(diethylamino)-2-oxo-1,2-dihydrobenzo[*b*][1,8]naphthyridin-3-yl)tetrahydrofuran-3-yl-2-cyanoethyldiisopropylphosphoramidite **12** (202 MHz; 298 K; $CDCl_3$).

Synthesis of oligonucleotides

Oligonucleotides were generated by synthesizing ABN phosphoramidite using published methods, and incorporated into DNA strands using solid-phase DNA synthesis performed by W. M. Keck Foundation (Yale School of Medicine). The HPLC-purified oligonucleotides were characterized by MALDI-TOF mass spectrometry and found to be consistent with calculated masses as shown in **Table S1**. Complementary DNA and unmodified DNA sequences were purchased from Integrated DNA Technologies, Inc. (San Diego, CA).

Table S1. ABN-modified oligonucleotides.

**Oligo number**	**Sequence name**	**Sequence (5′-3′)**	**Calculated mass [M+H]**	**Measured mass [M+H]**
ODN1	Hairpin	GCT TAG CAG GXT AGT GCT AAG C	6929.256	6933.297
ODN4	AXA	CGC AAX ATC G	3152.651	3152.006
ODN7	GXC	CGC AGX CTC G	3144.635	3143.611

Table S2. Complementary and unmodified oligonucleotides.

**Oligo number**	**Sequence name**	**Sequence (5′-3′)**
ODN2	HairpinA	GCT TAG CAC TA**A** CCT GCT AAG C
ODN3	HairpinG	GCT TAG CAC TA**G** CCT GCT AAG C
ODN5	TAT	CGA **TAT** TGC G
ODN6	TGT	CGA **TGT** TGC G
ODN8	GAC	CGA **GAC** TGC G
ODN9	GGC	CGA **GGC** TGC G
ODN10	HairpinT	GCT TAG CAG G**T**T AGT GCT AAG C
ODN11	ATA	CGC A**AT A**TC G
ODN12	GTC	CGC A**GT C**TC G
ODN13	HairpinC	GCT TAG CAG G**C**T AGT GCT AAG C
ODN14	ACA	CGC A**AC A**TC G
ODN15	GCC	CGC A**GC C**TC G

Photophysical measurements

Quantum yields measurements

UV-vis absorption and fluorescence measurements were performed using a quartz sub-micro cuvette (10 mm path length) purchased from Starnacell Inc. Steady-state emission scans were recorded using a PTI QuantaMaster QM-400 and absorbances were measured on a Shimadzu UV-1700 Pharmaspec spectrometer. Quantum yield were calculated using the comparative method of Williams *et al.* and measured in duplicate, at minimum.[7] Coumarin 153 in ethanol ($\phi_{F,ref}$ = 0.54; λ_{ex} = 420 nm) was used as a reference standard for all photophysical measurements. Samples were diluted such that all absorption measurements fell within a range of 0.01–0.1 to avoid inner filter effects. Subsequent dilutions were performed stepwise in order to obtain a minimum of six absorbance and emission spectra for quantum yield determinations. Quantum yields are calculated according to the equation:

$$\phi_F = \phi_{F,ref} \frac{\mathrm{I}}{I_{ref}} \frac{A_{ref}}{A} \frac{\eta^2}{\eta_{ref}^2}$$

Where ϕ_F is the quantum yield, I is the integrated fluorescence intensity, A is the absorbance at the excitation wavelength and η is the refractive index of the solvent.

Circular dichroism spectroscopy

To measure the structure of oligonucleotides, CD spectra were scanned using 5 µM samples (2.5 µM for Hairpin oligonucleotides with complementary strands) at 25 °C in a 0.20 cm quartz cuvette and an Aviv model 420 CD spectrophotometer. CD spectra were averaged from two scans ranging 350 nm to 200 nm, in 1 nm increments. Background 1X PBS spectra were subtracted and raw signal (θ_i) in mdeg was recorded. Duplex melting temperatures were determined by measuring the absorbance at 260 nm from 15 °C to 75 °C (20 °C to 85 °C for Hairpin oligonucleotides) in 1 °C increments. The data were normalized to a two-state model using the following equation:

$$\alpha_U = \frac{\theta_i - \theta_F}{\theta_U - \theta_F}$$

Where fraction unfolded (α_U) equals the difference between raw signal minus the signal of fully annealed duplex (θ_F), divided by the difference between fully denatured duplex signal (θ_U) and annealed duplex signal. The normalized values were fit to a logistic model using OriginLab to calculate the melting temperature according to the equation:

$$\alpha_U = \frac{1}{1 + e^{-k(T-T_m)}}$$

Where T is temperature, T_m is the duplex melting temperature, and k is the melting rate (not reported).

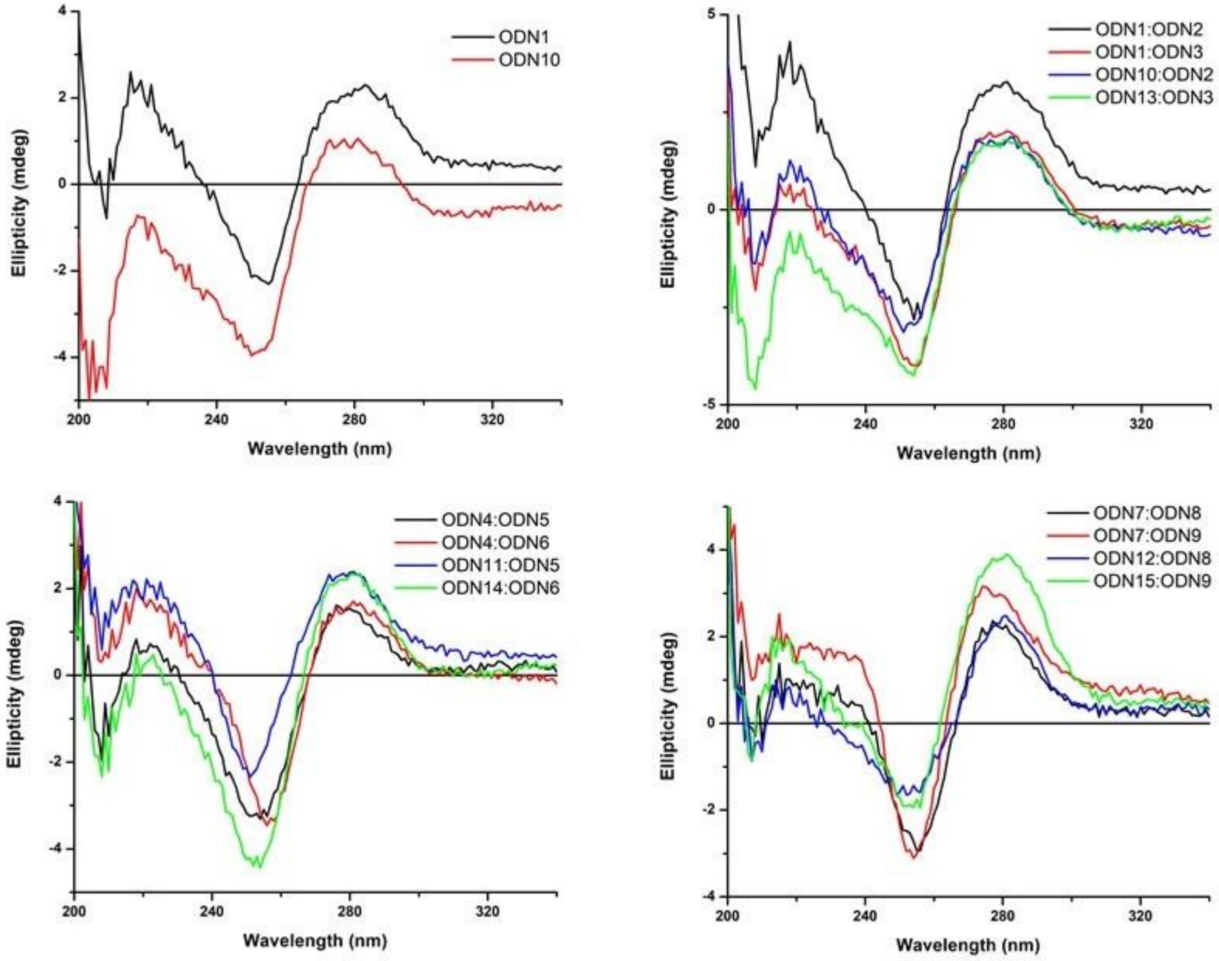

Figure S13. Circular dichroism spectra of ABN-modified and unmodified oligonucleotides in 1X PBS buffer (pH 7.4).

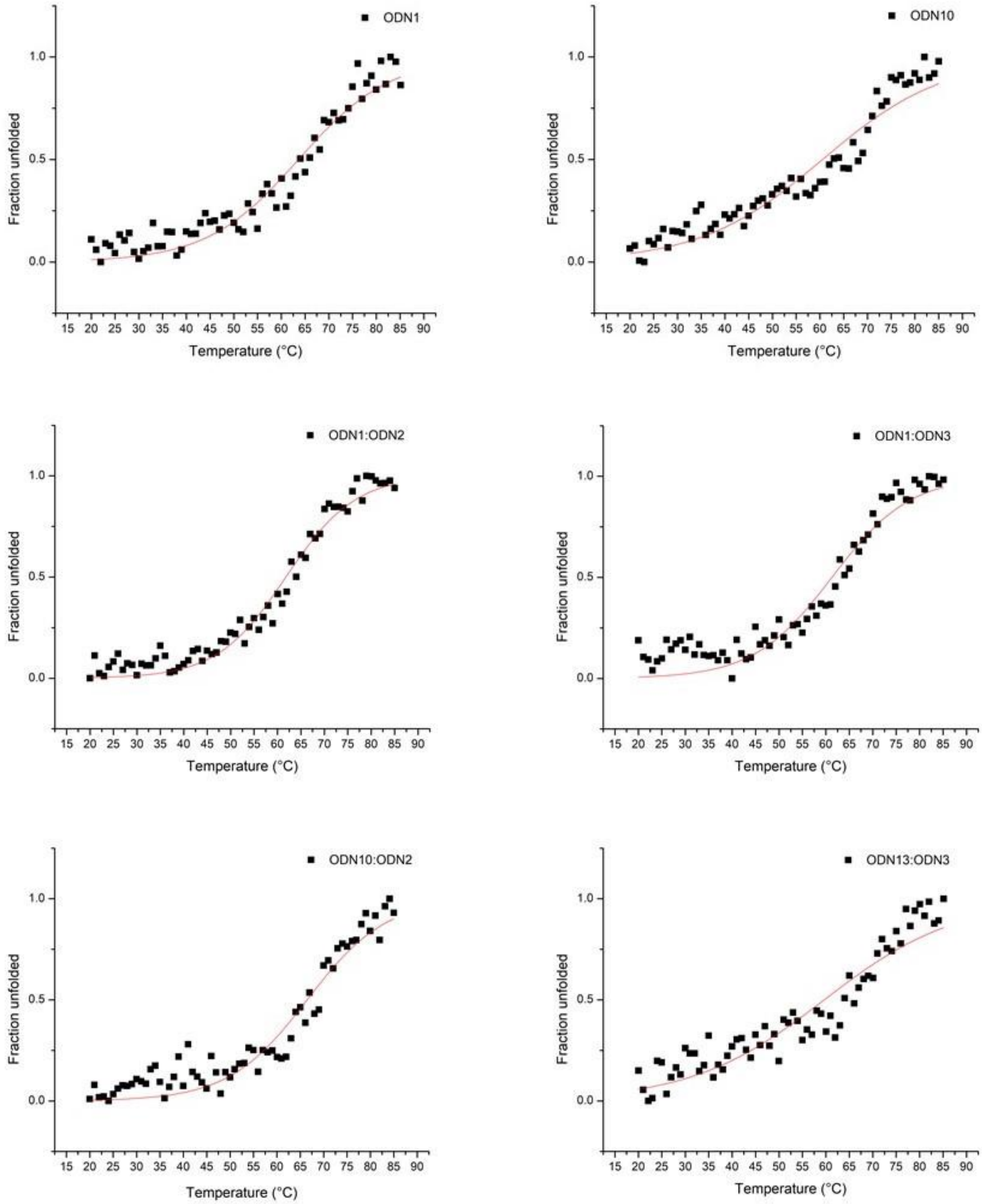

Figure S14. Melting temperature spectra of ABN-modified and unmodified oligonucleotides with hairpin structure in 1X PBS buffer (pH 7.4).

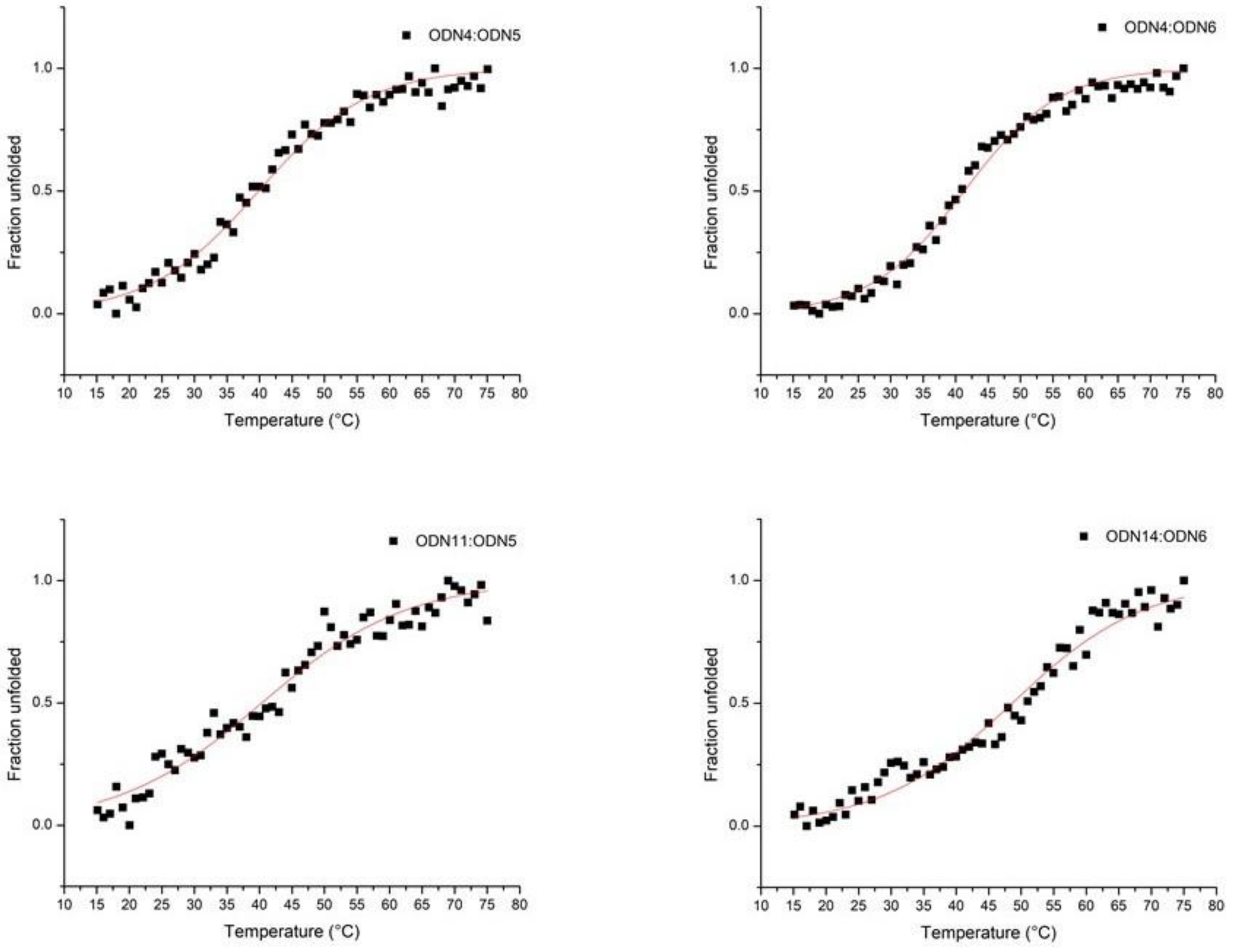

Figure S15. Melting temperature spectra of ABN-modified and unmodified oligonucleotides with two A flanking bases in 1X PBS buffer (pH 7.4).

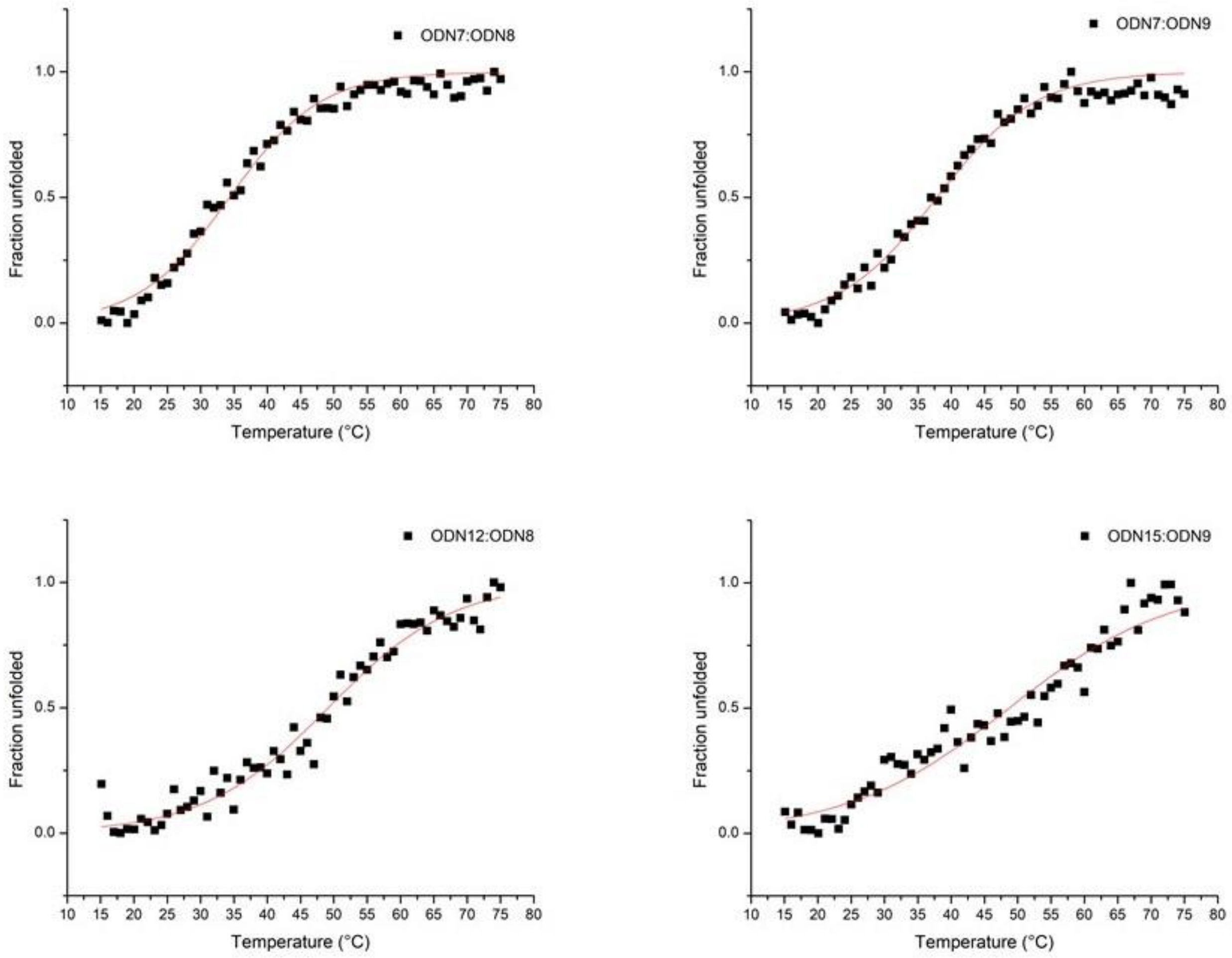

Figure S16. Melting temperature spectra of ABN-modified and unmodified oligonucleotides with G and C flanking bases in 1X PBS buffer (pH 7.4).

.

Additional figures

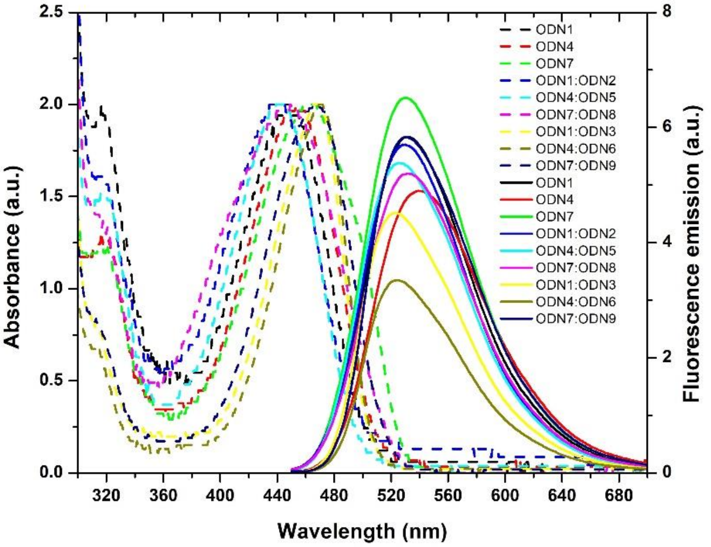

Figure S17. Absorbance (dashed line) and emission (solid line) spectra of ABN-modified oligonucleotides in 1X PBS buffer (pH 7.4).

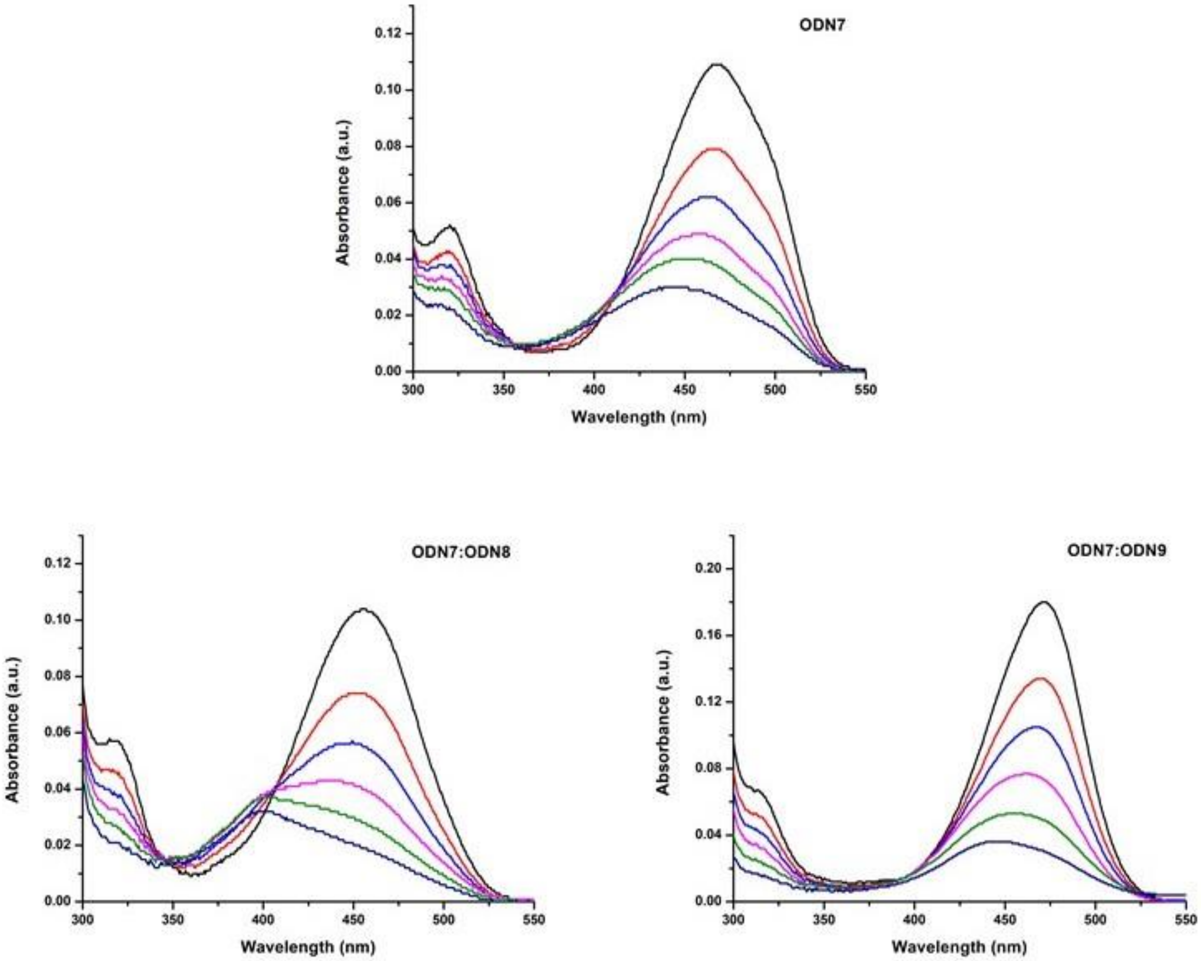

Figure S18. Absorption spectra of ODN7, ODN7:ODN8 and ODN7:ODN9 at different concentrations in 1X PBS buffer (pH 7.4).

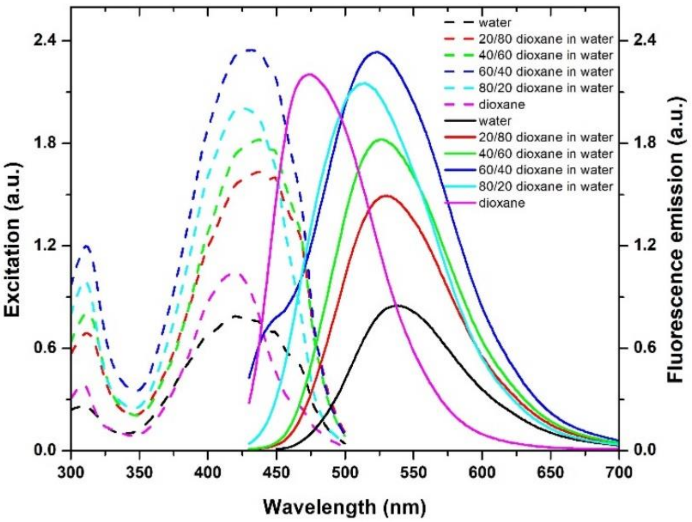

Figure S19. Excitation (dashed line) and emission (solid line) spectra of ABN in different ratios of dioxane to water. Excitation spectra were collected at λ_{em} of 530 nm, while emission spectra were collected at λ_{ex} of 420 nm.

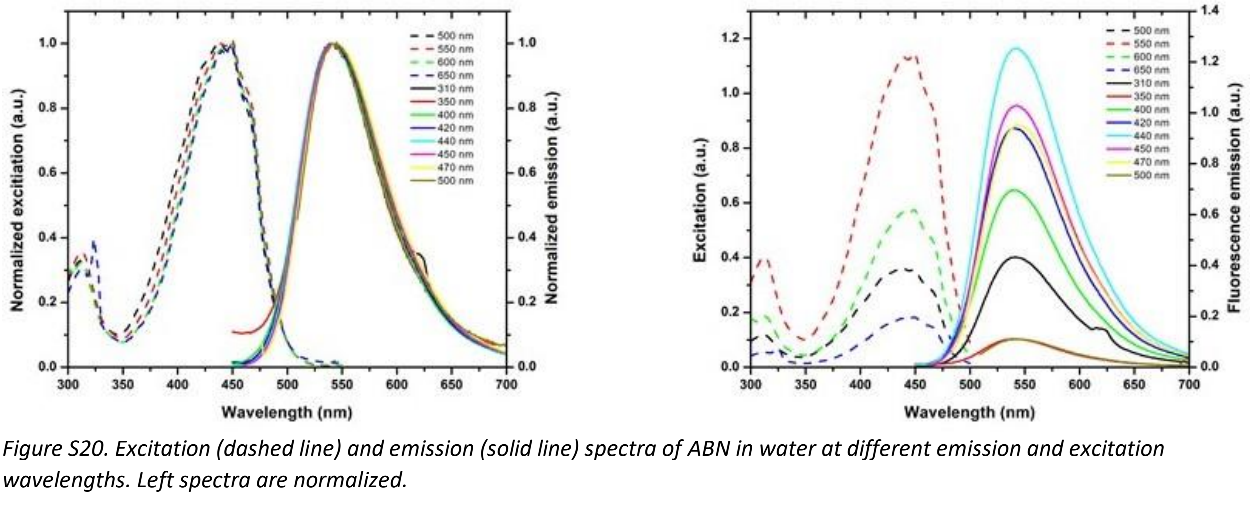

Figure S20. Excitation (dashed line) and emission (solid line) spectra of ABN in water at different emission and excitation wavelengths. Left spectra are normalized.

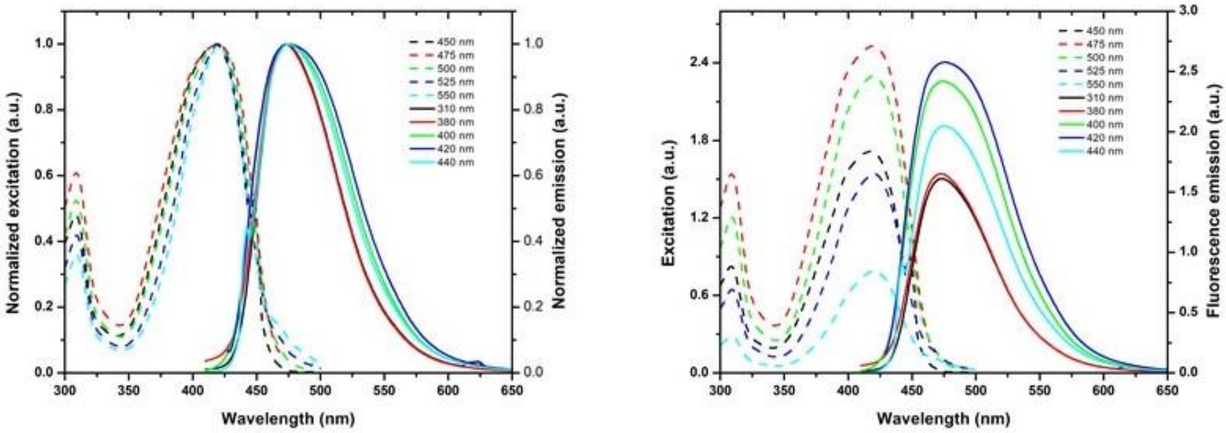

Figure S21. Excitation (dashed line) and emission (solid line) spectra of ABN in dioxane at different emission and excitation wavelengths. Left spectra are norrmalized.

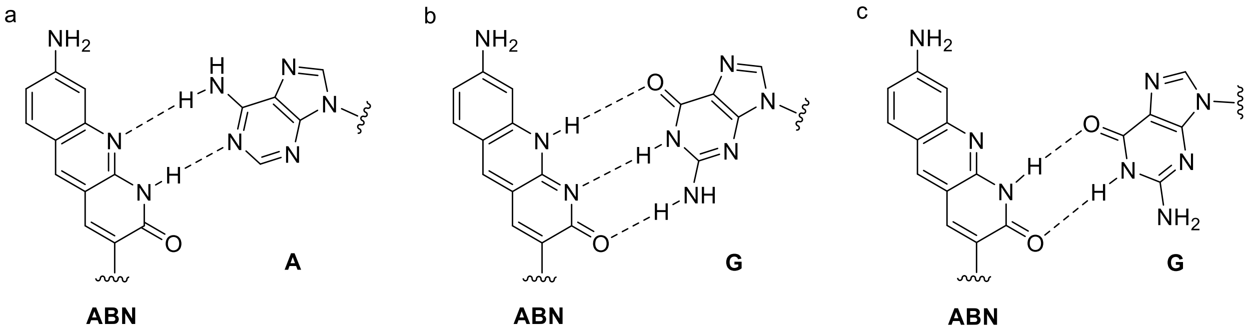

Figure S22. Possible hydrogen bonding pattern of ABN in duplex DNA. a) T:A Watson-Crick hydrogen bonding. b) Tautomeric base pairing, C:G Watson-Crick hydrogen bonding. c) T:G Wobble base pairing.

To view Movie S1, See DOI: 10.1039/d0sc03903a

Movie S1. Example movie showing fluorescence emission from surface-immobilised single-molecules of ABN undergoing 1P excitation.

1P single-molecule methods

Sample and slide preparation

ABN (10 mM, DMSO) was diluted into filtered (0.2 µm syringe filter, Whatman, 6780-1302) PBS (pH 7.4) to a working concentration of 100 nM. Borosilicate glass coverslips (VWR Int, 22 × 22 mm) were cleaned for 1 hour using an argon plasma (PDC-002, Harrick Plasma). Frame-Seal slide chambers (9 × 9 mm^2, Biorad, Hercules, CA) were affixed to the glass and the slides washed three times with filtered PBS (3 × 50 µL). The working solution of ABN (100 nM, 50 µL) was added to the slide and left for 3 minutes, allowing for some of the ABN molecules to adsorb to the plasma-cleaned glass surface. The excess solution containing unbound ABN was removed and the slide washed once with filtered PBS (1 × 50 µL) to yield spatially isolated single dyes on the glass surface.

Single-molecule imaging

Single-molecule fluorescence imaging of ABN was performed on a bespoke total internal reflection fluorescence microscope using a 488 nm continuous wavelength diode laser (iBeamSMART, Toptica, 200.46 W cm^{-2}). In steady-state ensemble measurements using a conventional spectrofluorometer, the ABN emission spectrum with excitation at of 488 nm is virtually identical to that with excitation at 440 nm, and a slightly lower Φ_{em} of 0.29 was recorded, as compared with Φ_{em} of 0.39 for excitation at 440 nm. The lower quantum yield is likely an artifact of the low absorptivity of ABN at this long wavelength. These data support that this single-molecule imaging is detecting the same species observed in steady-state ensemble measurements. The beam was circularly polarized using a wavelength specific quarter-wave plate and then expanded, collimated and aligned parallel to the optical axis at the edge of an objective lens (100× Plan Apo TIRF, NA 1.49 oil-immersion, Nikon Corporation) mounted on an inverted optical microscope (Ti2-Eclipse, Nikon Corporation). Fluorescence emission was collected by the same objective lens and separated from excitation light using a dichroic mirror (Di01-R405/488/561/635, Semrock) and passed through appropriate emission filters (BLP01-488R-25, FF01-520/44-25, Semrock). The fluorescence was then expanded (1.5 ×) and focused onto an electron-multiplying charge-coupled device (EMCCD, Evolve 512 Delta, Photometrics) with an electron multiplication gain of 250 ADU/photon operating in frame transfer mode. The effective pixel size was 107 nm. The instrument was automated using the open-source software micro-manager (v. 1.4) and the data displayed using the ImageJ software (v. 1.52d). Movies of single ABN emitters were collected for 150 frames at a rate of 10 fps.

Data analysis

A custom ImageJ macro was used to determine the total number of photons detected from single ABN emitters. The frames from single-molecule movies were summed and the pixel of peak intensity from each molecule was determined using the inbuilt 'Find Maxima' function (prominence threshold = 20,000). The single emitters were selected from a 6 × 6 region surrounding the pixel of peak intensity and the total integrated intensity of the region was measured. 6 × 6 pixel regions surrounding the segmented emitters were used to determine the local background integrated intensity. The background intensity was then subtracted from the total intensity of the peak to yield the total integrated signal above background. The intensity per ABN emitter was converted from counts to photons detected by dividing the integrated signal above background by the total gain of the EMCCD (35.7 ADU/photon).

2P single-molecule methods

Sample preparation

All experiments were performed with a solution of approximately 100 nM of ABN in TRIS buffer (20mM TRIS base) at a pH of 7.5. The molecules were initially dissolved in DMSO (at a concentration of 100 µM) and subsequently diluted in buffer. The TRIS buffer was prepared with ultrapure water (Direct Q3, Merck Millipore), TRIS-base (20 mM, Trizma base, BioUltra, Sigma-Aldrich) and 150 mM NaCl (analytical reagent grade, Fluka). The pH was adjusted to 7.5 with an HCl solution (Sigma-Aldrich). Prior to sample preparation, the buffer was filtered with activated charcoal (Darco, Fluca) and through a 0.2 µm filter (Milex, Merck).

Two-photon microscopy

The two-photon microscope is a homebuilt pulse-shaper assisted setup with a ultra-broadband Ti:Sa laser as light source, recently described.[8] The excitation source was a broadband Ti:Sapphire laser with a repetition rate of 80 MHz and a spectrum centered on 800 nm with a FWHM of 135 nm (Vitara UBB, Coherent). The compressed pulses from the oscillator had a duration of 15 fs. To compensate for dispersion in the objective the pulses were compressed using the MIIPS method with a pulse shaper (Biophotonics, MIIPS-Box 640).[9] The beam was subsequently focused onto the sample by a 60× water-immersion objective (UPlanSApo, Olympus). The pulses at the sample had a duration of 8 fs. The sample was placed on a cover slip (Menzel Gläser, Thermo Scientific) and the solution temperature was controlled (22 ± 1°C) with an incubator (Live Cell Instrument, CU-501). Sample fluorescence was collected by the same objective and transmitted through a dichroic mirror (Chroma 675dcspxr) and a short pass filter (Semrock FF01-650/SP), split by a polarising beamsplitter cube and detected by two avalanche photodiodes (MPD PDM 50c and MPD $PD-050-CTB). The signal was subsequently detected by a hardware correlator (ALV-7002, ALV GmbH).

Computational studies

NMR spectra were predicted for both the C-like and T-like tautomers using the B3LYP[10–12] density functional theory (DFT) method and the ab initio MP2[13,14] method in combination with cc-pVDZ, cc-pVTZ,[15,16] and pc-2[17] basis sets. To model relatively weak solvent interactions, the COSMO polarizable continuum method[18,19] was used with methanol as the solvent. All calculations were carried out with the *Gaussian 16* suite of programs.[20]

Benchmark analysis has shown that MP2 calculations tend to significantly outperform DFT methods in the prediction of NMR chemical shifts,[21] and so we report our MP2/cc-pVDZ/COSMO results for the proton and ^{13}C chemical shifts in Table S3. However, the qualitative results are consistent across all levels of theory and basis sets we examined. The patterns of the predicted spectra are shown in Figure S23, and the MP2/cc-pVDZ/COSMO optimized geometries (and the atom labeling scheme) are shown in Figure S24.

The calculated ^{13}C spectrum for the T-like tautomer is much more similar to the observed spectrum, showing in particular a separation among the peaks at d between 80 and 130 ppm (observed: 89, 103, 113, 116, 120; MP2 T-like: 80, 105, 112, 117, 119 ppm; MP2 C-like: 80, 94, 111, 112, 113 ppm), as well as other signatures. The calculations further support assignment of the observed tautomer to the T-like form by uniformly predicting the T-like form to be more stable than the C-like by 10–13 kcal mol^{-1} ($\Delta G =$ 10.3 kcal mol^{-1} for MP2/cc-pVDZ/COSMO energy + B3LYP/cc-pVDZ vibrational corrections). The HOMO and LUMO MOs of the C-like isomer are both predicted to have lower energies than in the T-like isomer, with larger decrease in LUMO energy more than the HOMO, resulting in a net redshift of the absorbance in the C-like relative to the T-like. Calculations were performed using a dimethylamino derivative of ABN instead of diethylamino groups in order to simplify the calculations.

Table S3. Calculated MP2/cc-pVDZ/COSMO chemical shifts (ppm) for proton and ^{13}C NMR spectra of both C-like and T-like tautomers, relative to TMS (calculated at the same level of theory)

C-like				T-like			
34-H	8.52	16-C	187.34	34-H	8.69	16-C	176.87
47-H	8.25	45-C	173.06	33-H	8.39	25-C	171.23
27-H	8.00	25-C	172.93	27-H	8.08	20-C	167.21
33-H	8.00	31-C	162.70	47-H	7.69	45-C	166.05
28-H	6.55	20-C	157.20	28-H	6.92	31-C	158.04
26-H	6.17	22-C	150.13	26-H	6.73	30-C	151.24
8-H	4.32	30-C	145.25	8-H	4.39	22-C	147.13
11-H	3.55	29-C	144.86	11-H	3.58	29-C	140.79
14-H	3.37	32-C	118.68	14-H	3.41	21-C	124.40
12-H	3.21	24-C	117.53	12-H	3.27	24-C	122.35
13-H	3.03	21-C	117.12	43-H	3.06	32-C	117.86
42-H	2.98	23-C	100.13	13-H	3.06	23-C	111.52
38-H	2.86	5-C	85.68	39-H	2.87	5-C	85.87
40-H	2.41	2-C	76.11	40-H	2.30	4-C	75.96
39-H	2.41	4-C	75.86	41-H	2.30	2-C	74.94
43-H	2.41	6-C	66.09	38-H	2.30	6-C	65.95
41-H	2.41	3-C	48.86	42-H	2.20	3-C	49.00
10-H	1.70	36-C	44.21	10-H	1.65	36-C	44.54
9-H	1.17	37-C	43.91	9-H	1.30	37-C	43.96
19-H	0.32			19-H	0.38		
18-H	0.10			18-H	0.14		

.

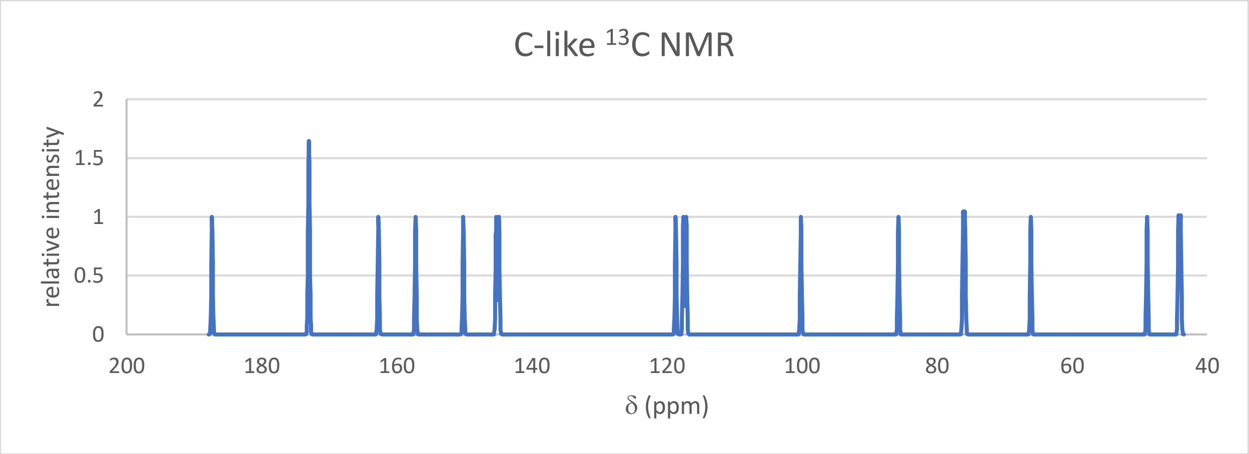

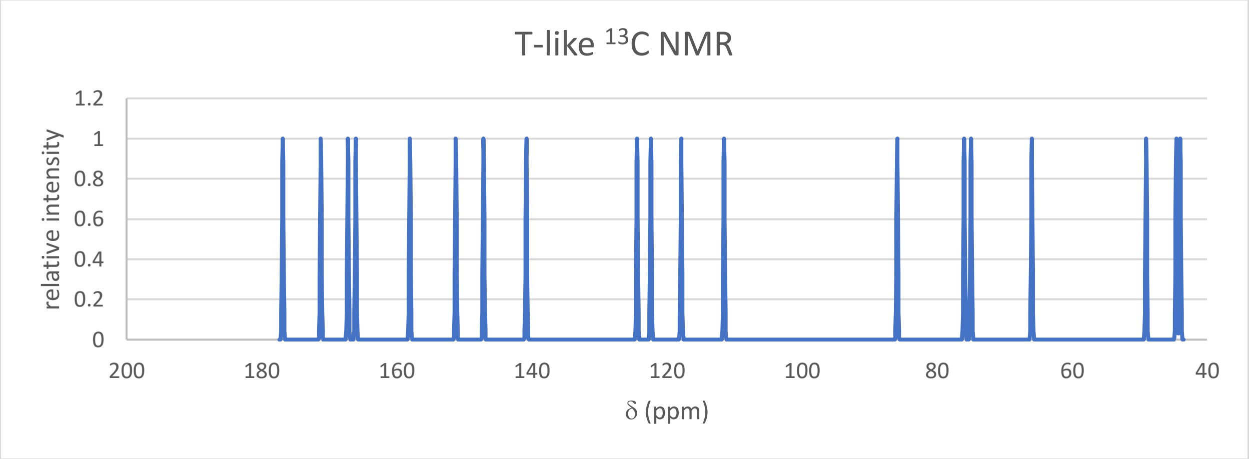

Figure S23. Predicted MP2/cc-pVDZ/COSMO ^{13}C NMR spectra.

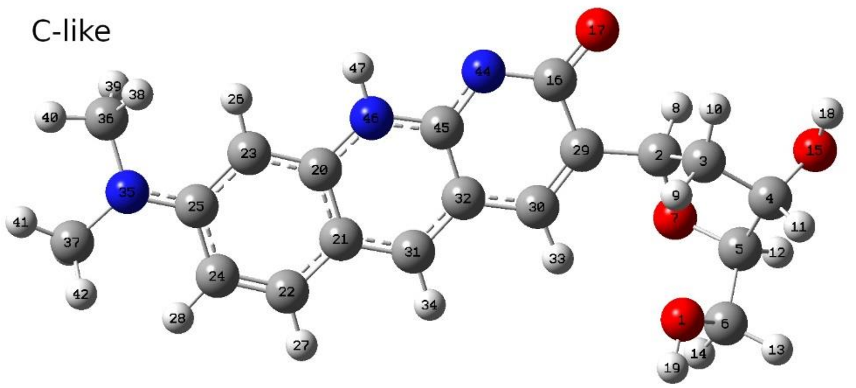

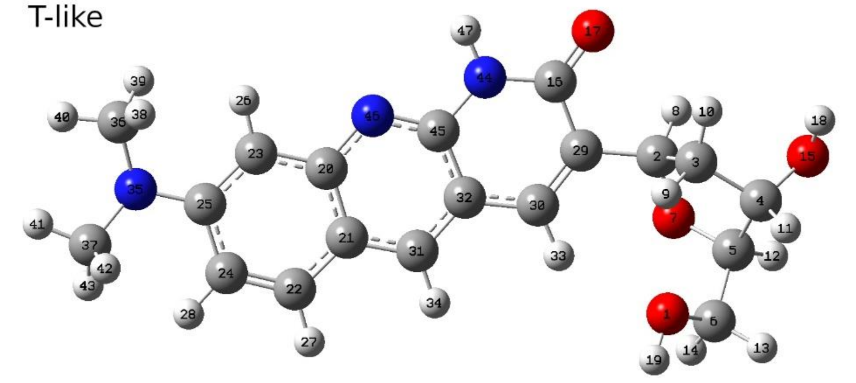

Figure S24. MP2/cc-pVDZ/COSMO optimized geometries of both tautomers of ABN.

References

(1) Meth-Cohn, O.; Rhouati, S.; Tarnowski, B.; Robinson, A. A Versatile New Synthesis of Quinolines and Related Fused Pyridines. Part 8.' Conversion of Anilides into 3-Substituted Quinolines and into Quinoxalines. *J Chem Soc Perkin Trans I* **1981**, *1*, 1537–1543.

(2) Gong, X.; Yang, H.; Liu, H.; Jiang, Y.; Zhao, Y.; Fu, H. Simple and Efficient Copper-Catalyzed Approach to 2,4-Disubstituted Imidazolones. *Org. Lett.* **2010**, *12* (14), 3128–3131. https://doi.org/10.1021/ol1008813.

(3) Liu, Y.-Y.; Thom, E.; Liebman, A. A. Coumarins via the Wittig Reaction. *J Heterocycl. Chem* **1979**, *16*, 799–801.

(4) Xia, M.; Hu, W.; Sun, S.; Yu, J.-T.; Cheng, J. The Dearomative Annulation between N-2-Pyridylamidine and CO2 toward Pyrido[1,2-a]-1,3,5-Triazin-4-Ones. *Org. Biomol. Chem.* **2017**, *15* (19), 4064–4067. https://doi.org/10.1039/C7OB00777A.

(5) Cheng, J. C. Y.; Hacksell, U.; Daves, G. D. Facile Synthesis of 2'-Deoxy-3'-Keto- and 2'-Deoxypseudouridine Derivatives and Analogues. Palladium(II)-Mediated Coupling Reactions of Furanoid Glycals. *J. Org. Chem.* **1986**, *51* (16), 3093–3098. https://doi.org/10.1021/jo00366a003.

(6) Evans, D. A.; Chapman, K. T.; Carreira, E. M. Directed Reduction of β-Hydroxy Ketones Employing Tetramethylammonium Triacetoxyborohydride. *J. Am. Chem. Soc.* **1988**, *110* (11), 3560–3578. https://doi.org/10.1021/ja00219a035.

(7) Williams, A. T. R.; Winfield, S. A.; Miller, J. N. Relative Fluorescence Quantum Yields Using a Computer-Controlled Luminescence Spectrometer. *Analyst* **1983**, *108* (1290), 1067–1071. https://doi.org/10.1039/AN9830801067.

(8) Fisher, R. S.; Nobis, D.; Füchtbauer, A. F.; Bood, M.; Grøtli, M.; Wilhelmsson, L. M.; Jones, A. C.; Magennis, S. W. Pulse-Shaped Two-Photon Excitation of a Fluorescent Base Analogue Approaches Single-Molecule Sensitivity. *Phys. Chem. Chem. Phys.* **2018**, *20* (45), 28487–28498. https://doi.org/10.1039/c8cp05496g.

(9) Lozovoy, V. V.; Pastirk, I.; Dantus, M. Multiphoton Intrapulse Interference. IV. Ultrashort Laser Pulse Spectral Phase Characterization and Compensation. *Opt Lett* **2004**, *29* (7), 775–777. https://doi.org/10.1364/OL.29.000775.

(10) Becke, A. D. Density-Functional Exchange-Energy Approximation with Correct Asymptotic Behavior. *Phys. Rev. A* **1988**, *38* (6), 3098–3100. https://doi.org/10.1103/PhysRevA.38.3098.

(11) Becke, A. D. A New Mixing of Hartree–Fock and Local Density-functional Theories. *J. Chem. Phys.* **1993**, *98* (2), 1372–1377. https://doi.org/10.1063/1.464304.

(12) Lee, C.; Yang, W.; Parr, R. G. Development of the Colle-Salvetti Correlation-Energy Formula into a Functional of the Electron Density. *Phys. Rev. B* **1988**, *37* (2), 785–789. https://doi.org/10.1103/PhysRevB.37.785.

(13) Frisch, M. J.; Head-Gordon, M.; Pople, J. A. A Direct MP2 Gradient Method. *Chem. Phys. Lett.* **1990**, *166* (3), 275–280. https://doi.org/10.1016/0009-2614(90)80029-D.

(14) Head-Gordon, M.; Pople, J. A.; Frisch, M. J. MP2 Energy Evaluation by Direct Methods. *Chem. Phys. Lett.* **1988**, *153* (6), 503–506. https://doi.org/10.1016/0009-2614(88)85250-3.

(15) Dunning, T. H. Gaussian Basis Sets for Use in Correlated Molecular Calculations. I. The Atoms Boron through Neon and Hydrogen. *J. Chem. Phys.* **1989**, *90* (2), 1007–1023. https://doi.org/10.1063/1.456153.

(16) Kendall, R. A.; Dunning, T. H.; Harrison, R. J. Electron Affinities of the First-row Atoms Revisited. Systematic Basis Sets and Wave Functions. *J. Chem. Phys.* **1992**, *96* (9), 6796–6806. https://doi.org/10.1063/1.462569.

(17) Jensen, F. Polarization Consistent Basis Sets: Principles. *J. Chem. Phys.* **2001**, *115* (20), 9113–9125. https://doi.org/10.1063/1.1413524.

(18) Barone, V.; Cossi, M. Quantum Calculation of Molecular Energies and Energy Gradients in Solution by a Conductor Solvent Model. *J. Phys. Chem. A* **1998**, *102* (11), 1995–2001. https://doi.org/10.1021/jp9716997.
(19) Cossi, M.; Rega, N.; Scalmani, G.; Barone, V. Energies, Structures, and Electronic Properties of Molecules in Solution with the C-PCM Solvation Model. *J. Comput. Chem.* **2003**, *24* (6), 669–681. https://doi.org/10.1002/jcc.10189.
(20) Frisch, M. J.; Trucks, G. W.; Schlegel, H. B.; Scuseria, G. E.; Robb, M. A.; Cheeseman, J. R.; Scalmani, G.; Barone, V.; Petersson, G. A.; Nakatsuji, H.; Li, X.; Caricato, M.; Marenich, A. V.; Bloino, J.; Janesko, B. G.; Gomperts, R.; Mennucci, B.; Hratchian, H. P.; Ortiz, J. V.; Izmaylov, A. F.; Sonnenberg, J. L.; Williams; Ding, F.; Lipparini, F.; Egidi, F.; Goings, J.; Peng, B.; Petrone, A.; Henderson, T.; Ranasinghe, D.; Zakrzewski, V. G.; Gao, J.; Rega, N.; Zheng, G.; Liang, W.; Hada, M.; Ehara, M.; Toyota, K.; Fukuda, R.; Hasegawa, J.; Ishida, M.; Nakajima, T.; Honda, Y.; Kitao, O.; Nakai, H.; Vreven, T.; Throssell, K.; Montgomery Jr., J. A.; Peralta, J. E.; Ogliaro, F.; Bearpark, M. J.; Heyd, J. J.; Brothers, E. N.; Kudin, K. N.; Staroverov, V. N.; Keith, T. A.; Kobayashi, R.; Normand, J.; Raghavachari, K.; Rendell, A. P.; Burant, J. C.; Iyengar, S. S.; Tomasi, J.; Cossi, M.; Millam, J. M.; Klene, M.; Adamo, C.; Cammi, R.; Ochterski, J. W.; Martin, R. L.; Morokuma, K.; Farkas, O.; Foresman, J. B.; Fox, D. J. *Gaussian 16 Rev. B.01*; Wallingford, CT, 2016.
(21) Flaig, D.; Maurer, M.; Hanni, M.; Braunger, K.; Kick, L.; Thubauville, M.; Ochsenfeld, C. Benchmarking Hydrogen and Carbon NMR Chemical Shifts at HF, DFT, and MP2 Levels. *J. Chem. Theory Comput.* **2014**, *10* (2), 572–578. https://doi.org/10.1021/ct400780f.
